# Alzheimer‐related protein APL‐1 modulates lifespan through heterochronic gene regulation in *Caenorhabditis elegans*

**DOI:** 10.1111/acel.12509

**Published:** 2016-08-24

**Authors:** Collin Y. Ewald, Vanessa Marfil, Chris Li

**Affiliations:** ^1^ Graduate CenterCity University of New YorkNew YorkNYUSA; ^2^ Department of BiologyCity College of New YorkNew YorkNYUSA; ^3^ Present address: Department of Health Sciences and TechnologyEidgenössische Technische Hochschule (ETH) Zürich8603SchwerzenbachSwitzerland

**Keywords:** Alzheimer's disease, APL‐1, APP, FOXO transcription factor DAF‐16, heat‐shock factor HSF‐1, heterochronic gene LIN‐14, lifespan, vitamin D‐like nuclear hormone receptor DAF‐12

## Abstract

Alzheimer's disease (AD) is an age‐associated disease. Mutations in the amyloid precursor protein (APP) may be causative or protective of AD. The presence of two functionally redundant *APP*‐like genes (APLP1/2) has made it difficult to unravel the biological function of APP during aging. The nematode *Caenorhabditis elegans* contains a single APP family member, *apl‐1*. Here, we assessed the function of APL‐1 on *C. elegans’* lifespan and found tissue‐specific effects on lifespan by overexpression of APL‐1. Overexpression of APL‐1 in neurons causes lifespan reduction, whereas overexpression of APL‐1 in the hypodermis causes lifespan extension by repressing the function of the heterochronic transcription factor LIN‐14 to preserve youthfulness. APL‐1 lifespan extension also requires signaling through the FOXO transcription factor DAF‐16, heat‐shock factor HSF‐1, and vitamin D‐like nuclear hormone receptor DAF‐12. We propose that reinforcing APL‐1 expression in the hypodermis preserves the regulation of heterochronic *lin‐14* gene network to improve maintenance of somatic tissues via DAF‐16/FOXO and HSF‐1 to promote healthy aging. Our work reveals a mechanistic link of how a conserved APP‐related protein modulates aging.

## Introduction

Alzheimer's disease (AD) is a neurodegenerative disorder affecting over five million Americans and is the sixth leading cause of death (Alzheimer's Association 2015). The etiology of Alzheimer's disease is unclear. The highest risk factor for developing Alzheimer's disease is age, whereby 96% of patients with AD develop the disease at 65 years or older (Alzheimer's Association 2015). The disease is characterized by the deposition of amyloid plaques in brain tissue; the major component of the plaques is the beta‐amyloid peptide, a cleavage product of the amyloid precursor protein (APP) (reviewed Selkoe & Hardy, 2016). Mutations in *APP* and in genes that produce the *presenilin* enzymes that are part of the γ‐secretase complex that cleaves APP are correlated with cases of familial Alzheimer's disease (Tanzi, 2012; reviewed Selkoe & Hardy, 2016).

In mammals, the functional analysis of APP is complicated by the presence of two APP‐related proteins, APLP1 and APLP2 (reviewed Shariati & De Strooper, 2013). Whereas knockouts of individual *APP* family members are viable, double knockouts of *APP* and *APLP2* or *APLP1* and *APLP2* result in postnatal lethality (von Koch *et al*., 1997; Herms *et al*., 2000, 2004), indicating an essential role for the APP family and functional overlap among family members that is not dependent on the beta‐amyloid peptide. Knockout of the entire APP gene family results not only in postnatal lethality, but also defects in neuronal migration and type II lissencephaly (Weyer *et al*., 2011). Interestingly, duplication of the *APP* gene has also been associated with families that develop early‐onset Alzheimer's disease (Cabrejo *et al*., 2006; Rovelet‐Lecrux *et al*., 2006; Sleegers *et al*., 2006). More strikingly, patients with Down syndrome, who have a trisomy of chromosome 21, have a high incidence of early‐onset Alzheimer's disease (Korbel *et al*., 2009; reviewed in Shariati & De Strooper, 2013). These findings suggest that higher levels of APP, whether it be higher levels of the beta‐amyloid peptide, the extracellular or cytoplasmic fragments, and/or full‐length APP, are correlated with early‐onset Alzheimer's disease. However, the cellular functions of APP and related proteins remain unclear.

The functions of genes correlated with Alzheimer's disease, including the presenilin genes, were first identified in genetic screens using the nematode *Caenorhabditis elegans* [reviewed (Ewald & Li, 2010)]. We have been examining the role of APL‐1, the *C. elegans* orthologue of mammalian APP. Similar to human APP, *C. elegans* APL‐1 is a single transmembrane‐spanning protein with a large extracellular and a small intracellular domain, both of which share sequence homology to human APP (Daigle & Li, 1993). Analogous to the postnatal lethality of *APP* family‐knockout mice, loss of *apl‐1* in *C. elegans* results in a completely penetrant larval lethality that is rescued by the reintroduction of either full‐length APL‐1 or only the extracellular domain of APL‐1 (Hornsten *et al*., 2007). This finding was later extended to mammals, whereby knockin of sAPPα rescued the postnatal lethality of the APP‐APLP2‐double‐knockout mice (Weyer *et al*., 2011). Furthermore, overexpression of APL‐1 in *C. elegans* also causes an early developmental lethality, but the lethality shows an incomplete penetrance that is correlated with higher levels of APL‐1 (Hornsten *et al*., 2007). Similarly, overexpression of human or mouse APP in mice causes early developmental lethality in the absence of amyloid plaques (Hsiao *et al*., 1995). This correlation suggests that findings in *C. elegans* can provide insights into the rudimentary conserved functions of APP (Ewald & Li, 2012).

Here, we examined the effects of overexpressing APL‐1 during adulthood on lifespan in *C. elegans*. Overexpressing APL‐1 in neurons shortens lifespan, whereas overexpressing APL‐1 in hypodermis extends lifespan. This lifespan extension is independent from longevity mediated by reduced insulin/IGF‐1 signaling (rIIS) or by reduced germ stem cell numbers (rGSC). However, genes that are essential for rIIS and rGSC longevity, such as the FOXO transcription factor DAF‐16, heat‐shock factor HSF‐1, and vitamin D‐like nuclear hormone receptor DAF‐12, are required for APL‐1‐dependent longevity, suggesting that APL‐1 interacts with these genes in parallel to rIIS and rGSC. We found that hypodermal APL‐1 represses the transcriptional function of the heterochronic transcription factor LIN‐14, which has been shown to regulate longevity through HSF‐1 and DAF‐16. Repressing LIN‐14 during aging is required for hypodermal‐expressed APL‐1 to extend lifespan and higher levels of LIN‐14 are sufficient to abrogate the APL‐1‐induced longevity. These findings suggest that APL‐1 promotes longevity by regulating heterochronic processes.

## Results

### Ectopic expression of APL‐1 during adulthood increases lifespan

**Figure 1:**
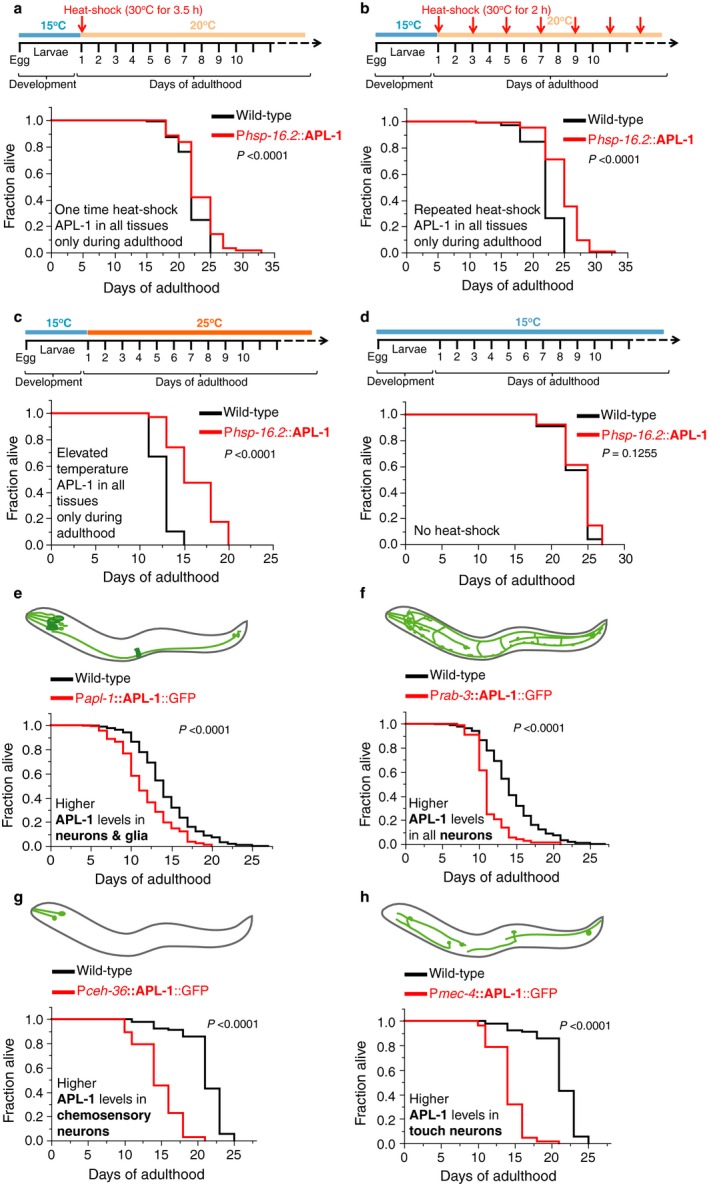
Overexpression of APL‐1 in all tissues during adulthood is sufficient to increase lifespan. (a–d) Wild‐type (N2) and transgenic *ynIs14* [P*hsp‐16.2*::APL‐1] animals that express APL‐1 driven by a heat‐shock promoter (P*hsp‐16.2*) were maintained at 15°C and shifted to different temperatures as indicated in the schematics. (a) A single heat shock during early adulthood was sufficient to increase the lifespan of *ynIs14* [P*hsp‐16.2*::APL‐1] animals. (b) Repeated heat shocks every other day during adulthood robustly increased the lifespan of *ynIs14* [P*hsp‐16.2*::APL‐1] animals. (c) Elevated temperatures were sufficient to drive APL‐1 expression from *hsp‐16.2* promoter (not shown; G. O'Connor, personal comm.) and increased lifespan of *ynIs14* [P*hsp‐16.2*::APL‐1] animals. (d) At lower temperatures, the *hsp‐16.2* promoter does not drive APL‐1 expression. The lifespan of *ynIs14* [P*hsp‐16.2*::APL‐1] animals was not extended. (e–h) Overexpression of APL‐1 with cell‐specific promoters and its own promoter in wild‐type animals. (e) Pan‐neuronal APL‐1 overexpression of *ynIs104* [P*rab‐3*::APL‐1::GFP] animals resulted in a shortened lifespan. (f) Transgenic *ynEx214* [P*ceh‐36*::APL‐1::GFP] animals that overexpress APL‐1 in chemosensory neurons showed a shortened lifespan. (g) Transgenic *ynIs113* [P*mec‐4*::APL‐1::GFP] animals that overexpress APL‐1 in touch mechanosensory neurons showed a shortened lifespan. (h) Driving APL‐1 overexpression from its endogenous promoter results in higher APL‐1 levels in neurons, glia cells, and vulva muscles [P*apl‐1*::APL‐1::GFP]. Transgenic *ynIs79* [P*apl‐1*::APL‐1::GFP] animals showed a shortened lifespan. For (a–d) Lifespan assays were run in parallel at the same time. *P‐*value was determined by log rank. Statistics and additional lifespan data are in Table S1. For (e–f). Lifespans are shown in cumulative form. For (e–h) Schematic representation of the expression pattern of APL‐1 with different promoters is from Ewald *et al*. (2012a>). Light green indicates neurons and dark green indicates other tissues. *P‐*value was determined by log rank. Statistics and additional lifespan data are in Tables S1 and S2.

High levels of APL‐1 cause larval lethality (Hornsten *et al*., 2007). To bypass the developmental effects of APL‐1 and to determine the role of APL‐1 during aging, we used transgenic animals (*ynIs14*) that have the *hsp‐16.2* promoter fused with an *apl‐1* cDNA [P*hsp‐16.2*::APL‐1; (Ewald *et al*., 2012a)] and induced APL‐1 expression in adults in all tissues via heat shock. Surprisingly, a single heat shock for 3.5 hours in day one adults mildly increased lifespan (5%; Fig. 1a). Moreover, two hours of heat shock administered every second day (Fig. 1b) or applying moderate heat continuously (Fig. 1c; 25°C) robustly increased lifespan (12–30%) of *ynIs14* [P*hsp‐16.2*::APL‐1] animals compared to wild‐type. As a control, the same animals were assayed in parallel at 15 °C, a temperature at which the *hsp‐16.2* promoter is not active, and no change in lifespan was observed (Fig. 1d; Table S1). These results suggest that inducing ubiquitous overexpression of APL‐1 exclusively during adulthood is sufficient to extend lifespan.

### Overexpressing APL‐1 in neurons shortens lifespan

The finding that ubiquitous adulthood expression of APL‐1 increased lifespan was surprising. To gain some mechanistic insights into how this lifespan extension is mediated, we investigated in which tissue *apl‐1* expression is necessary for lifespan extension. *C. elegans* expresses *apl‐1* strongly in neurons throughout its life cycle (Hornsten *et al*., 2007) and neurons regulate aging and longevity in a conserved spectrum of animals, including *C. elegans* (Kenyon, 2010). To investigate whether APL‐1 expressed in neurons increases lifespan, we examined transgenic animals carrying multicopy chromosomal arrays where *apl‐1* is driven by different promoters: its endogenous promoter, the pan‐neuronal *rab‐3* promoter that drives expression only in neurons, or the *ceh‐36* or *mec‐4* promoters that drive expression in a subset of neurons. Overexpression of APL‐1 with its own promoter, which includes strong expression in neurons and glial cells ([P*apl‐1*::*apl‐1*::GFP]; Fig. 1e; Table S2), shortened lifespan. Similarly, overexpressing APL‐1 in all neurons with the *rab‐3* (P*rab‐3*::APL‐1::GFP; Fig. 1f; Table S2), or a subset of sensory neurons (P*ceh‐36*::APL‐1::GFP; Fig. 1g and P*mec‐4*::APL‐1::GFP; Fig. 1h; Table S1), also shortened lifespan. Collectively, these results indicate that neuronal overexpression of APL‐1 shortens lifespan and is in stark contrast to the extended lifespan induced by heat‐shock overexpression of APL‐1 in all tissues, including neurons, in adults (Fig. 1). Therefore, we hypothesized that expression of APL‐1 in tissues other than neurons must activate a protective pathway to circumvent the detrimental effects of APL‐1 expression in neurons.

### Overexpression of APL‐1 in multiple tissue types, including neurons, increases lifespan

**Figure 2:**
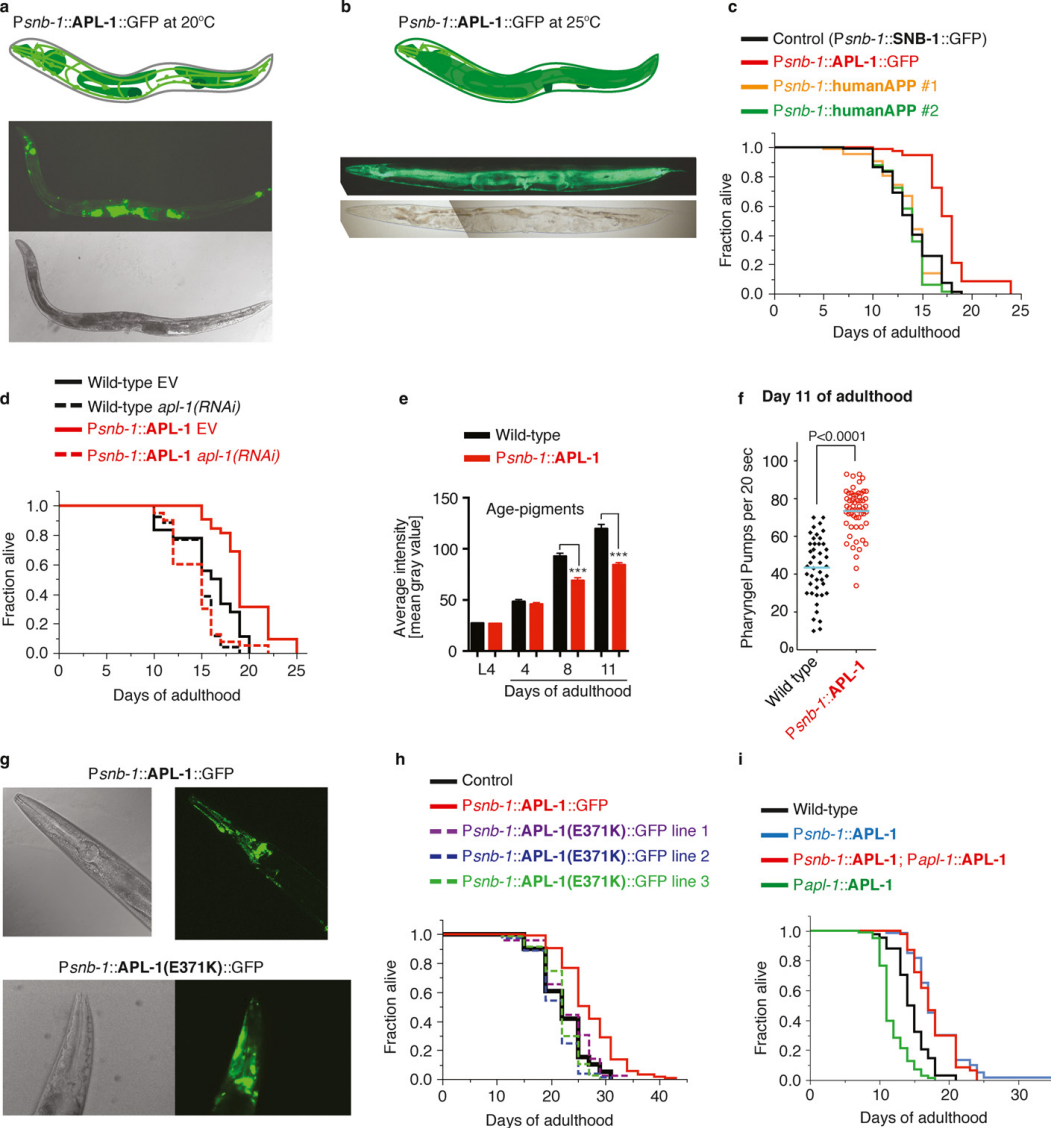
Overexpression of APL‐1 in neurons and other tissues extends lifespan. Using the *snb‐1* promoter, *apl‐1* [P*snb‐1*::APL‐1::GFP] is strongly expressed in neurons, pharynx, and somatic gonad and more weakly expressed in hypodermal cells during adulthood at the normal cultivation temperature of 20°C. (a, b) Top panel: Schematic representation of *apl‐1* expression. Middle panel: APL‐1::GFP expression driven by the *snb‐1* promoter. Bottom panel: Corresponding bright field. (a) Day 3 adults *lon‐2(e678) apl‐1(yn10) ynEx109* [P*snb‐1*::APL‐1::GFP] are shown with anterior to the left and ventral side down; animals were grown at 20°C. (b) At higher temperatures (25°C), the hypodermal APL‐1 expression becomes apparent in *ynIs109* [P*snb‐1*::APL‐1::GFP] animals. Day 1 *ynIs109* [P*snb‐1*::APL‐1::GFP] adults were shifted from 20°C to 25°C and day 3 adults are shown with anterior to the left and ventral side down. (c) Transgenic *ynIs109* [P*snb‐1*::APL‐1::GFP] showed extended lifespan compared to *jsIs1* [P*snb‐1*::SNB‐1::GFP] control animals. Driving human APP with the *snb‐1* promoter had no effect on lifespan. *P‐*value was determined by log rank. Statistics are in Table S3. (d) Knockdown of *apl‐1* by RNAi mildly shortened lifespan of wild‐type (N2) and suppressed longevity of *ynIs13* [P*snb‐1*::APL‐1] animals. *P‐*value was determined by log rank. Statistics are in Table S3. (e) Long‐lived *ynIs12* [P*snb‐1*::APL‐1] animals showed lower lipofuscin (age‐pigment) levels at days 8 and 11 compared to wild‐type (N2). Data were represented as mean ± SEM *P*‐values were determined by unpaired two‐tailed *t*‐test (***P < 0.0001). For scoring and additional strains, see Fig. S1a,b. (f) Long‐lived *ynIs13* [P*snb‐1*::APL‐1] animals showed pharyngeal pumping rates at day 11 compared to wild‐type (N2). Data represented as mean ± SEM *P*‐values were determined by unpaired two‐tailed *t*‐test. For additional strains and time points, see Fig. S1c–f. (g) Mutant or wild‐type APL‐1 expression driven with the *snb‐1* promoter shows similar GFP levels during adulthood at the normal cultivation temperature (20°C). Top panel shows head of a day 3 adult *lon‐2(e678) apl‐1(yn10) ynEx109* [P*snb‐1*::APL‐1::GFP] animal with bright field on the left and corresponding GFP on the right. Bottom panel shows head of a day 3 adult *ynEx236* [P*snb‐1*::APL‐1(E371K)::GFP] animal with bright field on the left and corresponding GFP on the right. (h) Transgenic animals that express a mutated APL‐1(E371K) with the *snb‐1* promoter failed to increase lifespan. Lines 1, 2, and 3 are *ynEx236, ynEx237,* and *ynEx238*, respectively. Statistics and additional lifespan data are in Table S1. (i) The double transgenic *ynIs12; ynIs86* animals, which carry two transgenes [P*snb‐1*::APL‐1] and [P*apl‐1*::APL‐1], that drive APL‐1 overexpression with its endogenous and *snb‐1* promoter, respectively, showed increased lifespan. Statistics and additional lifespan data are in Tables S2 and S3.

To identify the cells in which *apl‐1* expression counteracts the detrimental effects of pan‐neuronal *apl‐1* overexpression, we used the *snb‐1* promoter, which drives expression strongly in neurons and somatic gonad, but weakly in the hypodermis during adulthood (Fig. 2a; Ewald *et al*., 2012a>). APL‐1 expression was tracked by fusion to green fluorescent protein (GFP) at the C‐terminus (P*snb‐1*::APL‐1 cDNA::GFP), which did not interfere with rescuing *apl‐1(null)* larval lethality (Hornsten *et al*., 2007). The most prominent expression in *ynIs109* [P*snb‐1*::*apl‐1* cDNA::GFP] transgenic animals was in neurons and somatic gonad at 20°C (Fig. 2a). At 25°C, there were also high levels of APL‐1 expression in the hypodermis (Fig. 2b). *ynIs109* [P*snb‐1*::*apl‐1* cDNA::GFP] lived 28% longer than control animals at 20°C (Fig. 2c); this lifespan extension was not affected by the presence of the GFP tag, as animals carrying a transgene without a GFP tag (*ynIs12* [P*snb‐1*::*apl‐1* cDNA] and *ynIs13* [P*snb‐1*::*apl‐1* cDNA]) showed a comparable lifespan extension of 24% (average of eight individual trials) and 21% (average of five individual trials) longer than wild‐type, respectively (Table S2). Knockdown of *apl‐1* by RNA interference (RNAi) suppressed the increased longevity in *ynIs13* [P*snb‐1*::*apl‐1* cDNA] animals (Fig. 2d; Table S3), suggesting that the increased lifespan is specific to *apl‐1* expression. Furthermore, *ynIs13* [P*snb‐1*::*apl‐1* cDNA] animals showed a slowed aging process based on less accumulation of age‐pigments and enhanced muscle functions (Fig. 2e–f and S1). These results suggest that overexpressing APL‐1 in tissues other than neurons is beneficial for extending health and lifespan.

### Overexpression of functional APL‐1 is required to increase lifespan

There are several possible mechanisms by which P*snb‐1*::*apl‐1* cDNA overexpression could increase lifespan. For example, lowering food intake results in caloric restriction that increases lifespan (Kenyon, 2010). However, pharyngeal pumping rates, a measure of food intake, were unaltered in young transgenic APL‐1 animals (Fig. S1c). Another hallmark of caloric‐restricted animals is lower fat content (Heestand *et al*., 2013). The fat content of long‐lived P*snb‐1*::APL‐1 animals was not lower compared to wild‐type. Furthermore, the fat content of short‐lived *apl‐1(yn5)* and P*apl‐1*::APL‐1 animals was also lower compared to wild‐type animals (Fig. S2), suggesting that the effects of APL‐1 on fat content do not correlate with its effect on lifespan and are, therefore, uncoupled from longevity. Lifespan extension can also be induced in *C. elegans* by mobilizing the unfolded protein response (UPR) (Taylor & Dillin, 2013). APL‐1 is a glycosylated, transmembrane protein that is presumably processed in the endoplasmic reticulum (ER). Overexpression of APL‐1 could overload the ER causing ER stress, thereby activating the UPR. However, APL‐1 overexpression does not cause ER stress or induce the unfolded protein response (Fig. S3a–c). The increased lifespan, however, was dependent on a functional APL‐1 protein. The missense mutation *apl‐1(yn32)* causes an APL‐1 E371K substitution that presumably interferes with alpha secretase cleavage at the cell membrane. Expression of the *apl‐1(yn32)* transgene did not rescue the *apl‐1(null)* larval lethality (Hornsten *et al*., 2007; Hoopes *et al*., 2010), suggesting that APL‐1(*yn32)* is not sufficient for rescue. Animals carrying the P*snb‐1*::APL‐1(E371K)::GFP transgene (*ynEx236, 237, or 238*) showed no lifespan extension (Fig. 2h), suggesting that only functional APL‐1 can increase lifespan and that nonfunctional APL‐1 has no adverse effects. To further exclude that any protein structurally similar to APL‐1 (e.g., human APP) driven by the *snb‐1* promoter could extend lifespan, we measured the lifespan of animals carrying the P*snb‐1*::humanAPP transgene, which had no effect on lifespan (Fig. 2c; Table S3). Human APP is unable to rescue the *apl‐1(null)* larval lethality (Hornsten *et al*., 2007; Wiese *et al*., 2010), suggesting that human APP is not functional in *C. elegans*. Taken together, these results suggest that overexpression of functional APL‐1 increases lifespan.

### APL‐1 protein levels do not correlate with changes in lifespan

Another possibility for APL‐1 lifespan effects is that levels of the APL‐1 protein determine lifespan. However, levels of APL‐1 protein did not correlate with changes in lifespan, as *ynIs79* [P*apl‐1*::*apl‐1*::GFP] animals expressed the highest levels of APL‐1::GFP, but showed a shortened lifespan (Figs. 1e and S3d–f). Instead, we postulate that the increased lifespan is context dependent; that is, where APL‐1 is overexpressed determines the APL‐1 effect on longevity. Hence, as long as APL‐1 is overexpressed in the relevant tissues, lifespan will be increased. We tested this model by mating short‐lived *ynIs86* [P*apl‐1*::APL‐1] transgenic animals that show a 177‐fold increase in APL‐1 protein (Hornsten *et al*., 2007) with long‐lived *ynIs12* [P*snb‐1*::APL‐1] transgenic animals that show a 71‐fold increase in APL‐1 protein (Hornsten *et al*., 2007). The resultant double transgenic strain showed a 199‐fold increase in APL‐1 levels compared to wild‐type and was long‐lived (Figs. 2i and S3d–e; Table S3), demonstrating that the tissue in which *apl‐1* is expressed and not the overall levels of APL‐1 protein determines lifespan.

### Overexpression of APL‐1 in hypodermal cells is sufficient to increase lifespan

**Figure 3:**
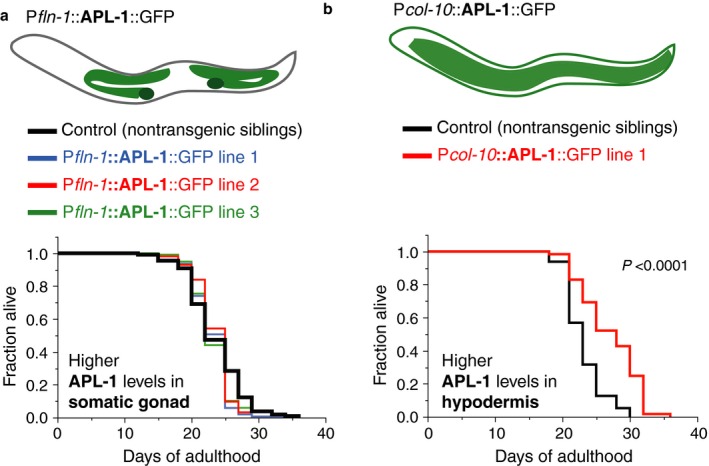
Ectopic hypodermal APL‐1 expression extends lifespan. (a) Driving APL‐1 expression in the somatic gonad with the *fln‐1* promoter was not sufficient to increase lifespan. Lines 1, 2, and 3 are *ynEx242, ynEx243,* and *ynEx244*, respectively. (b) Driving APL‐1 expression in the hypodermis with the *col‐10* promoter was sufficient to increase lifespan. Line 1 is *ynEx234*. For (a, b) *P‐*value was determined by log rank. Statistics and additional lifespan data are in Table S1.

The long‐lived *ynIs109* [P*snb‐1*::APL‐1::GFP] transgenic animals showed prominent APL‐1::GFP expression in neurons and the somatic gonad and slightly weaker expression in the hypodermis (Fig. 2). The somatic gonad has previously been shown to regulate lifespan via hormonal signaling (Kenyon, 2010; Antebi, 2013). However, driving APL‐1 expression exclusively in the somatic gonad or in combination with neuronal APL‐1 did not alter lifespan (Fig. 3a; Table S1). Lastly, we tested whether hypodermal APL‐1 expression could increase lifespan. We generated transgenic animals that overexpress APL‐1 exclusively in the hypodermis (*ynEx234* [P*col‐10*::APL‐1::GFP]). These transgenic lines showed an 11–22% increase in lifespan and fully recapitulated the increase in longevity seen in *ynIs109* [P*snb‐1*::APL‐1::GFP] animals (Fig 3b; Tables S1 and S4). Hence, *apl‐1* overexpression in the hypodermis is critical for the APL‐1‐dependent increase in lifespan.

### Overexpression of the extracellular domain of APL‐1 is sufficient to affect lifespan in a tissue‐dependent manner

**Figure 4:**
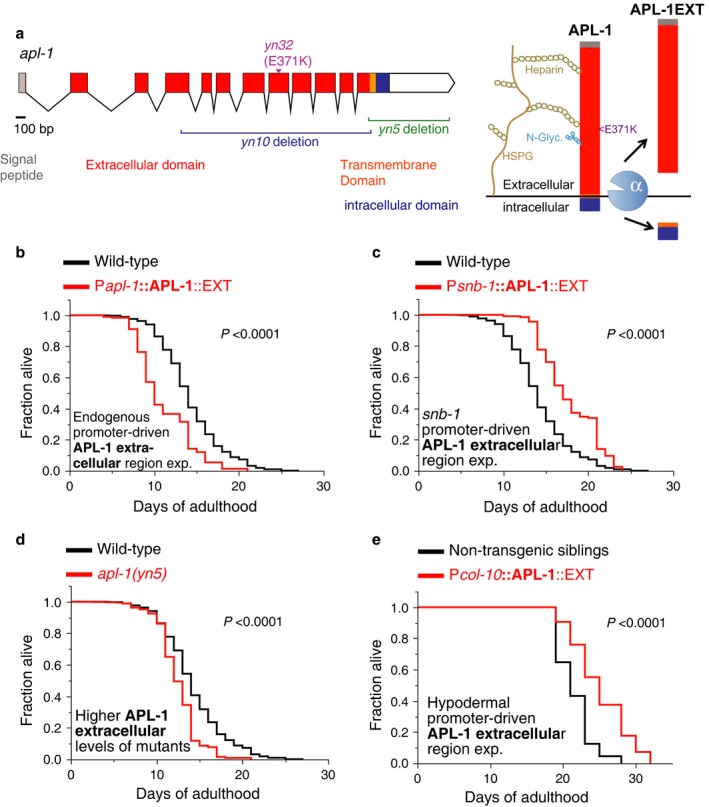
The extracellular domain of APL‐1 modulates lifespan depending on the tissues in which it is expressed. (a) Schematic of genomic region (left panel) and model of APL‐1 cleavage (right panel). α = alpha secretase, HSPG = heparan sulfate proteoglycans, N‐Glyc. = N‐glycosylation. The extracellular domain of APL‐1 has two potential HS binding domains. (b) Transgenic *ynIs71* [P*apl‐1*::APL‐1EXT] animals that overexpress the extracellular domain of APL‐1 (APL‐1EXT) driven by its endogenous promoter showed a shortened lifespan. (c) Transgenic *ynIs105* [P*snb‐1*::APL‐1EXT] animals that overexpress the extracellular domain of APL‐1 (APL‐1EXT) driven by the *snb‐1* promoter showed an extended lifespan. (d) *apl‐1(yn5)* mutants, which contain higher levels of the extracellular domain of APL‐1 compared to wild‐type (Fig. S3d,e), showed a shortened lifespan. (e) Transgenic *ynEx241* [P*col‐10*::APL‐1EXT] animals that overexpress the extracellular domain of APL‐1 (APL‐1EXT) in the hypodermis showed an extended lifespan. For (b‐d) Lifespans are shown in cumulative form. Statistics and additional lifespan data are in Table S2.

Similar to other APP family members, *C. elegans* APL‐1 is a single transmembrane‐spanning protein with a large extracellular and a small intracellular domain (Fig. 4a; Daigle & Li, 1993). The extracellular domain of APL‐1 (APL‐1EXT), which is analogous to sAPL‐1, is necessary and sufficient to rescue the *apl‐1(null)* lethality (Hornsten *et al*., 2007; Wiese *et al*., 2010). Furthermore, the *apl‐1(yn5)* allele completely lacks the genomic region encoding the transmembrane and intracellular domains, but is viable (Hornsten *et al*., 2007). To determine whether the extracellular domain of APL‐1 modulates lifespan, we examined *apl‐1(yn5)* mutants and transgenic animals that overexpress the extracellular domain of APL‐1 (APL‐1EXT) in a wild‐type background. Both *apl‐1(yn5)* mutants and transgenic animals that overexpressed APL‐1EXT from the *apl‐1* promoter (*ynIs71* [P*apl‐1*::APL‐1EXT]) showed shortened lifespans, similar to the shortened lifespan when full‐length APL‐1 was overexpressed (Fig. 4b,c). By contrast, when APL‐1EXT expression was driven with the *snb‐1* promoter (*ynIs105* [P*snb‐1*::APL‐1EXT]), the animals showed an extended lifespan compared to wild‐type (Fig. 4d). Furthermore, expressing APL‐1EXT in the hypodermis increased lifespan (*ynEx241* [P*col‐10*::APL‐1EXT]; Fig. 4e; Table S1). By contrast, expressing APL‐1EXT in the hypodermis is not sufficient to rescue the lethality seen in *apl‐1(yn10)* null alleles (0/3 lines rescued). Thus, the extracellular domain of APL‐1 modulates aging in a tissue‐specific manner.

### Extended lifespan of P*snb‐1*::APL‐1 transgenic animals requires *daf‐16* and *daf‐12* activity

**Figure 5:**
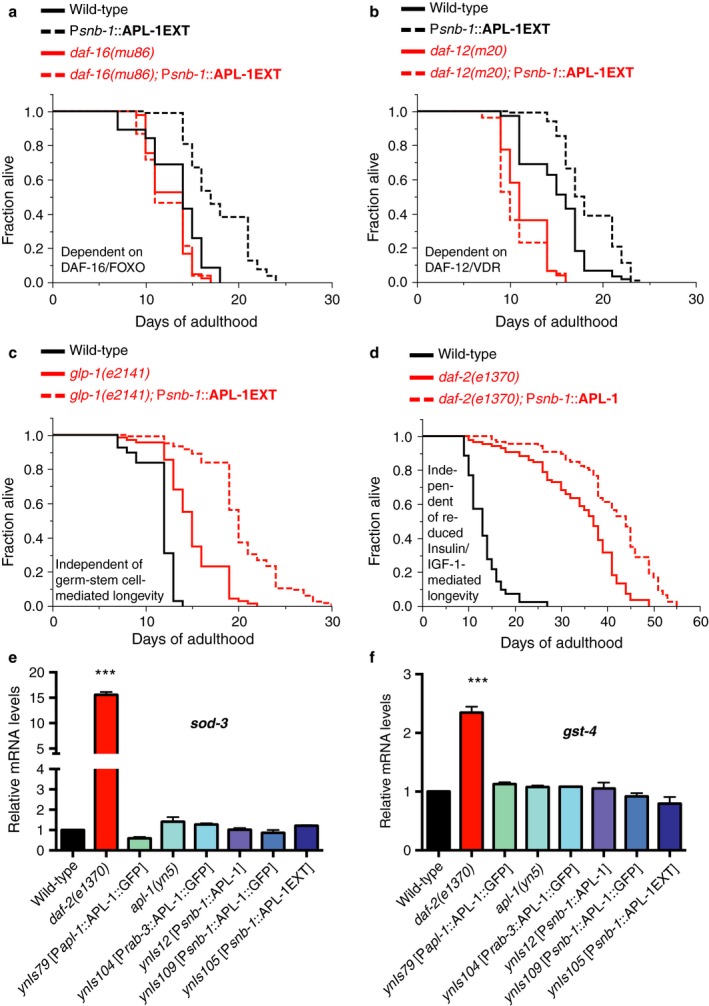
The APL‐1‐induced longevity requires DAF‐16 and DAF‐12. (a) The longevity of transgenic *ynIs105* [P*snb‐1*::APL‐1EXT] animals was suppressed by a null mutation in *daf‐16*, which encodes a FOXO transcription factor. (b) The longevity of transgenic *ynIs105* [P*snb‐1*::APL‐1EXT] animals was suppressed by a mutation in *daf‐12*, which encodes a vitamin D‐like nuclear hormone receptor (VDR). (c) The longevity of transgenic *ynIs105* [P*snb‐1*::APL‐1EXT] animals was additive to the longevity of animals with reduced germ stem cell numbers *(glp‐1(e2141))*. (d) The longevity of transgenic *ynIs12* [P*snb‐1*::APL‐1] animals was additive to the longevity of animals with reduced Insulin/IGF‐1 signaling *(daf‐2(e1370))*. (e–f) mRNA levels of canonical DAF‐16 target genes (*sod‐3* and *gst‐4*) under reduced insulin/IGF‐1 conditions are not altered in animals that overexpress APL‐1. Three independent trials of *N* > 1000 of mixed‐stage animals per trial were analyzed. Data are represented as mean ± SEM *P‐*value ***<0.0001 relative to wild‐type (N2) was determined by one‐sample *t*‐test, two‐tailed, hypothetical mean of 1. For (a–d) *P‐*value was determined by log rank. Statistics and additional lifespan data are in Tables S2 and S3.

We have previously demonstrated that overexpression of APL‐1EXT slows developmental progression via the FOXO transcription factor DAF‐16 and vitamin D‐like nuclear hormone receptor DAF‐12 VDR (Ewald *et al*., 2012b>). Both DAF‐16 FOXO and DAF‐12 VDR activities are essential to mobilize mechanisms that extend lifespan (Kenyon, 2010; Antebi, 2013). Thus, we investigated whether overexpression of APL‐1 modulates lifespan via *daf‐16* and *daf‐12*. The longevity of *ynIs12* [P*snb‐1*::*apl‐1* cDNA], *ynIs109* [P*snb‐1*::*apl‐1* cDNA::GFP], and *ynIs105* [P*snb‐1*::APL‐1EXT] was completely abolished in *daf‐16(null)* and *daf‐12* mutants (Fig. 5a,b; Tables S1 and S3), suggesting that activity of DAF‐16 FOXO and DAF‐12 VDR is required to mediate longevity by APL‐1 overexpression. Furthermore, we assessed whether other known transcriptional regulators essential for extending lifespan are required for the P*snb‐1*::APL‐1EXT‐mediated longevity (Fig. S3g). We found that *hsf‐1* (heat‐shock factor 1) was completely and *skn‐1* (Nrf1,2,3 homologue) was partially required for the lifespan extension of *ynIs105* [P*snb‐1*::APL‐1EXT] animals (Table S3).

Many reproductive and metabolic disruptions increase lifespan. For instance, loss of germline stem cells, such as with a *glp‐1(e2141)* mutation, or reducing insulin/IGF‐1 signaling, such as with a *daf‐2(e1370)* mutation, extends lifespan via *daf‐16* and/or *daf‐12* (Kenyon, 2010). We investigated how APL‐1EXT released from hypodermal cells acts to increase lifespan. When germline stem cells were removed with a *glp‐1* mutation, the longevity of *ynIs105* [P*snb‐1*::APL‐1EXT] animals was further extended (Fig. 5c; Table S3). In addition, RNAi of *kri‐1* and *tcer‐1*, genes required for the *glp‐1* mutant‐mediated longevity, did not suppress the longevity of *ynIs105* [P*snb‐1*::APL‐1EXT] animals (Table S3), suggesting that APL‐1 overexpression increases lifespan in a pathway independent of the longevity pathway mediated by the loss of germline stem cells. Similarly, the longevity of *ynIs12* [P*snb‐1*::APL‐1] was further extended when insulin/IGF‐1 signaling was reduced (rIIs; Fig. 5d; Tables S1 and S3), again suggesting that the APL‐1‐mediated longevity is independent of the reduced rIIs‐mediated longevity pathway. Furthermore, mRNA levels of *sod‐3* and *gst‐4*, genes that are transcriptionally upregulated under reduced insulin/IGF‐1 conditions (Murphy *et al*., 2003; Ewald *et al*., 2015), were not altered when APL‐1 was overexpressed (Fig. 5e–f). Taken together, these results suggest that hypodermal APL‐1 interacts with DAF‐16 and DAF‐12 in pathways parallel to the insulin/IGF‐1 and germline stem cells pathways to increase lifespan.

### Hypodermal APL‐1 longevity is mediated by the heterochronic transcription factor LIN‐14

**Figure 6:**
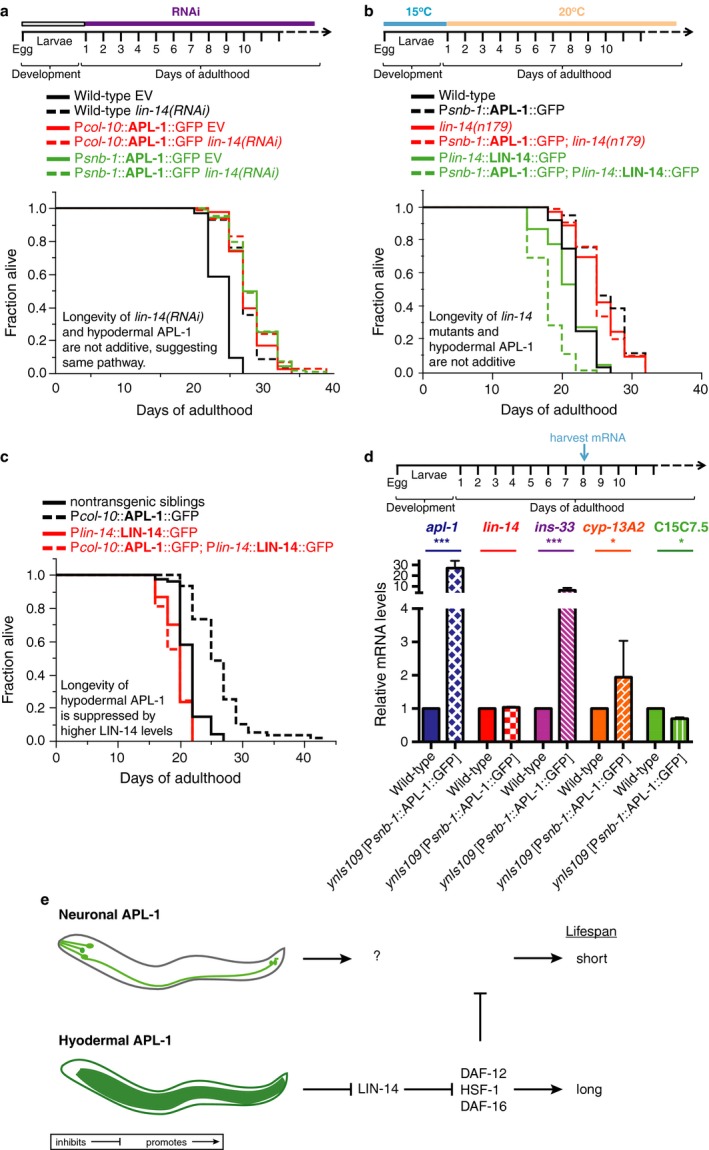
The APL‐1‐dependent longevity is mediated via the heterochronic transcription factor LIN‐14. (a) The longevity of transgenic *ynEx234* [P*col‐10*::APL‐1::GFP] and *ynIs109* [P*snb‐1*::APL‐1::GFP] animals was not additive to the longevity of animals treated with *lin‐14* RNAi. (b) Higher LIN‐14 protein levels of *zaIs2* [LIN‐14::GFP] transgenic animals that contain the *lin‐4* microRNA binding sites in the 3′UTR were sufficient to suppress the longevity of *ynIs109* [P*snb‐1*::APL‐1::GFP] animals. The longevity of transgenic *ynIs109* [P*snb‐1*::APL‐1::GFP] animals was not additive to the longevity of animals carrying a mutation in the *lin‐14* gene *(lin‐14(n179*)). (c) Higher LIN‐14 protein levels suppressed the longevity of *ynEx234* [P*col‐10*::APL‐1::GFP] animals. (d) mRNA levels of canonical LIN‐14 target genes were altered in day 8 of transgenic *ynIs109* [P*snb‐1*::APL‐1::GFP] adults compared to wild‐type. Three independent trials of *N* = 200 day 8 adult animals per trial were analyzed. Data are represented as mean ± SEM *P‐*value *<0.05 and ***<0.0001 relative to wild‐type (N2) was determined by one‐sample *t*‐test, two‐tailed, hypothetical mean of 1. (e) Model of how APL‐1 modulates lifespan. Neuronal APL‐1 overexpression shortens lifespan through an unknown mechanism (upper panel). Hypodermal APL‐1 overexpression represses LIN‐14 accumulation during aging, thereby releasing the inhibition of DAF‐12, HSF‐1, and DAF‐16 activity to promote longevity (lower panel). Repression of LIN‐14 and activation of DAF‐12, HSF‐1, and DAF‐16 are sufficient to protect against the adverse effects of higher APL‐1 levels in neurons. For (a–c) *P‐*value was determined by log rank. Statistics and additional lifespan data are in Table S4.

Recently, *daf‐16* FOXO and *daf‐12* VDR activities have been shown to form a regulatory circuit with *lin‐14* to regulate longevity (Shen *et al*., 2012). LIN‐14 is a heterochronic transcription factor that controls the transition from the first to second larval stage during development (Hristova *et al*., 2005). At the first larval stage (L1), LIN‐14 protein levels are high, thereby regulating genes that retain cells in the L1 stage (Ambros & Horvitz, 1984, 1987). During the first to second larval stage transition and later in development, the microRNA *lin‐4* prevents *lin‐14* translation by an RNA–RNA interaction (Lee *et al*., 1993). However, this inhibition is gradually lost during aging, when *lin‐4* miRNA levels decrease, thereby allowing LIN‐14 protein levels to increase (Boehm & Slack, 2005; Ibáñez‐Ventoso *et al*., 2006; Ibáñez‐Ventoso & Driscoll, 2009). Interestingly, knocking down *lin‐14* levels by RNAi in adults or by a temperature‐sensitive *lin‐14* mutation during the larval‐to‐adult transition is sufficient to increase lifespan via *daf‐16* FOXO and heat‐shock factor HSF‐1 (Boehm & Slack, 2005). Thus, we investigated whether the P*snb‐1*::APL‐1 transgene expression mediates lifespan extension by acting in the same pathway as LIN‐14. The P*snb‐1*::APL‐1 transgene‐mediated longevity was not further increased by either RNAi knockdown of *lin‐14* or by a *lin‐14(n179)* mutation (Fig. 6a,b; Table S4). Furthermore, RNAi knockdown of *lin‐14* was not additive to the longevity of hypodermal APL‐1 (*ynEx234* [P*col‐10*::APL‐1::GFP; Fig. 6a; Table S4], suggesting that hypodermal APL‐1 and *lin‐14* act in the same pathway to mediate longevity. Conversely, overexpressing LIN‐14 protein in *ynIs109* [P*snb‐1*::APL‐1::GFP] abolished the P*snb‐1*::APL‐1‐mediated longevity (Fig. 6b; Table S4), suggesting that APL‐1 activity represses the activity of LIN‐14. Interestingly, increasing LIN‐14 protein levels in *ynIs109* [P*snb‐1*::APL‐1::GFP] animals results in a shortened lifespan (Fig. 6b; Table S4). We hypothesize that repression of *lin‐14* mobilizes a protective response against the detrimental effects of neuronal APL‐1. We tested this hypothesis by increasing levels of LIN‐14 in hypodermal APL‐1 overexpression lines and found the suppression of hypodermal APL‐1‐induced longevity, but no lifespan reduction compared to wild‐type (Fig. 6c; Table S4). To determine whether hypodermal APL‐1 represses LIN‐14 activity during aging, we collected mRNA of postreproductive 8‐day‐old adult animals and measured the expression levels of three canonical LIN‐14 target genes, *cyp‐13*,* ins‐33*, and *C15C7.5*. During the first larval stage when LIN‐14 expression is high, *C15C7.5* is upregulated, while *cyp‐13* and *ins‐33* are downregulated (Hristova *et al*., 2005). If APL‐1 acts to repress LIN‐14 activity, we predicted that genes that are normally repressed by LIN‐14 are upregulated and genes that are normally upregulated by LIN‐14 are downregulated. Consistent with this hypothesis, *cyp‐13* and *ins‐33* were upregulated and *C15C7.5* gene was downregulated in *ynIs109* [P*snb‐1*::APL‐1::GFP] animals compared to wild‐type at day 8 of adulthood (Fig. 6d), suggesting that hypodermal APL‐1 is repressing LIN‐14 activity during aging. Taken together, we propose that hypodermal APL‐1 represses LIN‐14 levels during aging, which prolongs lifespan via *hsf‐1, daf‐12,* and *daf‐16* (Fig. 6e).

## Discussion

Mammalian APP is expressed in all tissue types (Tanzi *et al*., 1987), but an unresolved question remains as to whether APP has a similar function in all cell types or whether its functions are context dependent. As this question is difficult to address in mammalian systems, we turned to *C. elegans* where the use of cell‐specific promoters is routine. Because higher levels of APP have been correlated with Alzheimer's disease and Down syndrome, we asked whether levels of APL‐1, the *C. elegans* APP‐related protein, have an effect on lifespan. Longevity in any organism is the result of a combination of environmental effects and genetic factors. *C. elegans* is constantly monitoring its surroundings and integrating this information to regulate its metabolic and reproductive processes, which ultimately determine the animal's lifespan. Several tissues, such as neurons, intestine, and gonad, have been implicated in regulating lifespan in *C. elegans* (Kenyon, 2010). For instance, ablation of certain amphidial sensory neurons can either lead to lifespan enhancement or reduction; similarly, ablation of germline precursor cells leads to an increased lifespan. These effects on lifespan are dependent in DAF‐16 FOXO and DAF‐12 VDR activity (Kenyon, 2010).

The effects of APL‐1 overexpression on lifespan were context dependent (Fig. 6e). Overexpressing *apl‐1* under its own promoter caused accelerated aging and lifespan reduction. By contrast, ubiquitous expression of *apl‐1* in adults caused lifespan extension. These results suggest that where APL‐1 is expressed impacts its effects on lifespan. Because *apl‐1* expression in neurons, but not in hypodermal cells, is sufficient to rescue the lethality of *apl‐1* null mutants, we hypothesized that neuronal *apl‐1* expression was responsible for lifespan extension. Unexpectedly, the converse results were found; namely, *apl‐1* overexpression only in neurons led to lifespan reduction. However, *apl‐1* overexpression in hypodermal tissue is critical for lifespan extension and this lifespan extension is additive to the longevity induced by reduction in insulin signaling or by the removal of germline stem cells. Furthermore, we showed that expression of only the extracellular domain of APL‐1 in these tissues was sufficient to cause these lifespan effects and lifespan extension is dependent on the activity of *daf‐16* FOXO and DAF‐12 VDR. By contrast, hypodermal expression of *apl‐1* or APL‐1EXT is not sufficient to rescue the lethality of *apl‐1* null mutants. Hence, the target of neuronal release of sAPL‐1 vs. hypodermal release of sAPL‐1 appears different. We hypothesize that migration of sAPL‐1 is limited or confined by its local environment. For instance, sAPL‐1 has two heparin binding domains (Fig. 4a), suggesting that the secreted APL‐1 could interact locally with the extracellular matrix to limit its diffusion. Deleting the heparin binding domains of APL‐1EXT resulted in a failure to rescue the *apl‐1* null lethality (unpublished observation). Furthermore, overexpression of APL‐1EXT lacking the heparin binding domains had no effect on lifespan (Table S3), suggesting that interactions between sAPL‐1 and the extracellular matrix are essential for APL‐1 function. Collectively, these results indicate that APL‐1 has multiple functions: expression of *apl‐1* in neurons is required for viability, whereas expression of *apl‐1* in hypodermal cells regulates longevity.

The lifespan effects due to *apl‐1* overexpression were not dependent on downregulating metabolic pathways via insulin/IGF‐1 signaling or decreasing numbers of germline stem cells, but were influenced by the heterochronic pathway. *apl‐1* has been previously linked to the *let‐7* microRNA and heterochronic regulatory network (Niwa *et al*., 2008), which is composed of a regulatory cascade of protein regulators and microRNAs (miRNAs) that coordinate the onset of stereotypical stage‐specific developmental events (Ambros & Horvitz, 1987; Rougvie & Moss, 2013). In particular, while levels of the LIN‐14 transcription factor are negatively regulated by the *lin‐4* miRNA to allow transition from the first to the second larval stage (Lee *et al*., 1993), levels of *lin‐4* miRNA decrease with age, allowing LIN‐14 levels to increase, thereby limiting the lifespan of *C. elegans* (Ibáñez‐Ventoso & Driscoll, 2009). We propose that hypodermal *apl‐1* expression represses levels of LIN‐14, resulting in an increased lifespan relative to wild‐type animals (Fig. 6e).

In *C. elegans*, maintenance of somatic tissues is lost during the first day of adulthood (Labbadia & Morimoto, 2014). This is consistent with the disposable soma theory that predicts that resources are allocated from the somatic tissue to the germ line (Kirkwood & Holliday, 1979), which happens when animals become reproductive during the L4‐to‐adult transition. Interestingly, using a transcriptional reporter of the endogenous promoter of *apl‐1* expressing GFP, *apl‐1* has been shown by Niwa and colleagues to be transiently expressed under the control of *let‐7* miRNA network during the L4‐to‐adult transition in the hypodermal seam cells (Niwa *et al*., 2008). However, this hypodermal APL‐1 expression is lost in adulthood and we show here that ectopically expressing APL‐1 during adulthood in the hypodermis leads to increased health‐ and lifespan. We speculate that reinforcing APL‐1 expression in the hypodermis is a reprogramming of developmental networks via heterochronic LIN‐14 to improve the maintenance of somatic tissues via DAF‐16/FOXO and HSF‐1 that leads to improved organismal lifespan. The implication of an APP‐related protein functioning in a miRNA and heterochronic gene network to promote healthy aging might point toward a possible entry to use miRNAs as a therapeutic intervention to modulate age‐related consequences of APP function.

## Material and methods

### Strains

*Caenorhabditis elegans* strains were grown and maintained on MYOB or NGM plates containing OP50 *Escherichia coli* bacteria at 20°C, unless noted. All mutations are described in WormBase (www.wormbase.org) and include LGI: *daf‐16(mu86)*; LGII: *tcer‐1(tm1452)*; LGIII: *daf‐2(e1370)*,* glp‐1(e2141)*; LGX: *daf‐12(m20)*,* lon‐2(e678), apl‐1(yn5* and *yn10)* (Hornsten *et al*., 2007), *dpy‐8(e130)*, and *lin‐14(n179)*. Construction of the APL‐1 transgenes and the resulting transgenic lines are described (Hornsten *et al*., 2007) and (Ewald *et al*., 2012a>), except as indicated. Nonintegrated transgenic lines used were *ynEx30* [P*apl‐1*::APL‐1, *sur‐5*::GFP], *ynEx65* [P*snb‐1*::APL‐1(*yn5*)(cDNA), *sur‐5*::GFP], *ynEx93* [P*rab‐3*::APL‐1(cDNA)::GFP, pRF4 *rol‐6(su1006gf)*], *ynEx109* [P*snb‐1*::APL‐1(cDNA)::GFP], *ynEx212* [P*mec‐4*::APL‐1 (cDNA)::GFP, P*myo‐3*::mCherry], *ynEx213* [P*mec‐4*::APL‐1 (cDNA)::GFP, P*myo‐3*::mCherry], *ynEx214* [P*ceh‐36*::APL‐1 (cDNA)::GFP, P*myo‐3*::mCherry], *ynEx236* [P*snb‐1*::APL‐1(E371K)::GFP, P*myo‐3*::mCherry], *ynEx237* [P*snb‐1*::APL‐1(E371K)::GFP, P*myo‐3*::mCherry], *ynEx238* [P*snb‐1*::APL‐1(E371K)::GFP, P*myo‐3*::mCherry], *ynEx242* [P*fln‐1*::APL‐1::GFP, P*myo‐3*::mCherry]), *ynEx243* [P*fln‐1*::APL‐1::GFP, P*myo‐3*::mCherry], *ynEx244* [P*fln‐1*::APL‐1::GFP, P*myo‐3*::mCherry], *ynEx234* [P*col‐10*::APL‐1 (cDNA)::GFP, P*myo‐3*::mCherry], *ynEx235* [P*col‐10*::APL‐1 (cDNA)::GFP, P*myo‐3*::mCherry], *ynEx239* [P*col‐10*::APL‐1EXT (cDNA), *sur‐5*::GFP], *ynEx240* [P*col‐10*::APL‐1EXT (cDNA), *sur‐5*::GFP], and *ynEx241* [P*col‐10*::APL‐1EXT (cDNA), *sur‐5*::GFP]. Integrated transgenic lines used were *ynIs100* [P*apl‐1*::APL‐1(*yn32*), pRF4 *rol‐6(su1006gf)*], *ynIs106* [P*apl‐1*::APL‐1(*yn32 yn5*)], *ynIs14* [P*hsp‐16.2*::APL‐1 (cDNA), *lin‐15B*(+)],P*myo‐2*::GFP), *ynIs113* [P*mec‐4*::APL‐1 (cDNA)::GFP, P*myo‐3*::mCherry]**,** *jsIs1* [P*snb‐1*:: SNB‐1::GFP, pRF4 *rol‐6(su1006gf)*], *dvEx371* [P*snb‐1*::humanAPP751 (cDNA), P*mtl‐2*::GFP], *dvEx372* [P*snb‐1*::humanAPP751 (cDNA), P*mtl‐2*::GFP], *zaIs2* [LIN‐14::GFP, *rol‐6(su1006)*] (Hong *et al*., 2000; Olsson‐Carter & Slack, 2010); LGI: *ynIs109* [P*snb‐1*::APL‐1(cDNA)::GFP]; LGII: *ynIs105* [P*snb‐1*::APL‐1(*yn5*)(cDNA), *sur‐5*::GFP]; LGIII: *jsIs682* [P*rab‐3*::GFP::RAB‐3], *ynIs12* [P*snb‐1*::APL‐1(cDNA), *lin‐15B*(+)]; LGIV: *vsIs13* (*lin‐11*::*pes‐10*::GFP, *lin‐15*(+)), *ynIs104* [P*rab‐3*::APL‐1(cDNA)::GFP, P*myo‐2*::GFP], *zIs356* [P*daf‐16*::DAF‐16::GFP, pRF4 *rol‐6(su1006gf)*]; LGV: *ynIs13* [P*snb‐1*::APL‐1(cDNA), *lin‐15B*(+)], *ynIs71* [P*apl‐1*::APL‐1(*yn5*), *sur‐5*::GFP], *ynIs79* [P*apl‐1*::APL‐1::GFP], *ynIs100* [P*apl‐1*::APL‐1(*yn32*)::GFP, pRF4 *rol‐6(su1006gf)*]; and LGX: *dvIs62* (P*snb‐1*::humanTDP‐43, P*mtl‐2*::GFP), *ynIs86* [P*apl‐1*::APL‐1, *sur‐5*::GFP], *ynIs91* [P*rab‐3*::APL‐1 (cDNA), pRF4 *rol‐6(su1006gf)*], *ynIs107* [P*apl‐1*::APL‐1(*yn32* D342C/S362C)::GFP, P*myo‐2*::GFP], and *ynIs108* [P*apl‐1*::APL‐1(ΔH, ΔE2)::GFP; *sur‐5*::GFP].

### Generating transgene constructs

To eliminate the heparin sulfate binding domains (H) of APL‐1, the coding region for REEGSPCKWTHSVR, corresponding to amino acids 96–106, was deleted from the pAPL‐1ΔE2::GFP construct (Hornsten *et al*., 2007), which already had the heparin binding domain within E2 deleted, to generate the P*apl‐1*::APL‐1(ΔH, ΔE2)::GFP construct with both heparin sulfate binding domains deleted.

### RNA interference

RNAi clones were picked from the Ahringer or Vidal libraries. Cultures were grown overnight in LB with 12.5 μg/mL tetracycline and 100 μg/mL ampicillin, diluted to an OD600 of 1, and induced with 1 mM IPTG. This culture was seeded onto NGM agar plates containing tetracycline, ampicillin, and additional IPTG. Empty vector (EV) plasmid pL4440 was used as control.

### Lifespan assays

Because our laboratory uses a different growing media (MYOB) than other laboratories, which normally use (NGM), and wild‐type shows a different mean lifespan of 14 days on MYOB vs. 21 days on NGM (Keightley & Caballero, 1997), we performed most experiments on both media as indicated (Tables S1–S4). Lifespan results were similar on MYOB or on NGM. Note that the wild‐type N2 var. Bristol strain has been maintained for many generations in different laboratories and can have mean lifespans ranging from 12 to 17 days at 20°C (Gems & Riddle, 2000). N2 animals from another laboratory (kindly provided by Cathy Savage‐Dunn) showed a similar lifespan as our N2 stock on MYOB plates in four independent trials (198/398 animals). To determine whether the laboratory N2 animals had a longer lifespan on NGM plates, N2 animals were grown on MYOB until the L4 stage and then transferred onto NGM plates for lifespan analysis; these animals showed a mean lifespan of 18.62 ± 0.47 days (60/111 animals). These results indicate that the N2 mean lifespan of 14 days reported here is due to the different composition of the MYOB plates compared to NGM plates.

For all lifespan trials, NGM: L4 animals were picked onto fresh OP50 plates, and then day one adults were placed on either OP50 or RNAi plates containing 50 μm 5‐fluoro‐2′‐deoxyuridine (FUdR), unless otherwise indicated, as described in Ewald *et al*. (2015). All lifespan trials on MYOB were performed without FUdR. All strains were grown for at least two generations at 20°C, except for *daf‐2(e1370)* and *glp‐1(e2141)* animals, which were raised at 15°C. All adult lifespans [except *glp‐1(e2141)*] were measured starting from the L4 stage at 20°C. Animals carrying the temperature‐sensitive *glp‐1(e2141)* allele and appropriate control animals were shifted from 15°C to 25°C at the L1 stage and lifespan assays were performed at 25°C.

### Pharyngeal pumping assays

The pharyngeal pumping rate was determined by the number of pumps per 20 s; measurements were only taken when worms were on bacteria and pumped at a constant rate without any pauses as described (Ewald *et al*., 2012b>).

### Autofluorescence assays

L4 animals were picked and scored at appropriate times. Animals were placed on an agar pad containing a drop of 10 mm NaN_3_. Pictures were taken with a Hamamatsu ORCA‐ER digital camera on a Zeiss Axioplan microscope. The area directly behind the last bulb of the pharynx was photographed using Openlab 3.1.3 software (Improvision, Coventry, United Kingdom) with the same settings for all animals and conditions. The autofluorescence intensities were derived by selecting a defined area of the worm in the pictures and computing the ‘mean gray value’ by ImageJ software (NIH).

### Western blot analysis

Preparations of animal lysates and Western blots were performed as described (Hornsten *et al*., 2007). Protein levels were normalized to levels of actin (~40 kDa) for each strain, so that similar amounts of total protein were loaded for each strain. For each blot, the blot was first probed with an actin monoclonal antibody (JLA20 at 1:2000; Developmental Studies Hybridoma Bank, Iowa City, Iowa, United States; secondary antibody anti‐mouse‐goat‐HRP at 1:1000), washed three times with TAE, and then re‐probed with an anti‐APL‐1 antiserum against the extracellular domain of APL‐1 (Hornsten *et al*., 2007). Relative protein levels were determined by relative intensity to wild‐type (N2) using NIH Image J Gel analyzer.

### Quantitative real‐time polymerase chain reaction (qRT‐PCR) assays

Mixed‐stage animals were harvested (>1000 animals; three independent trials; Figs. 5e–f and S3b,c,f). For adulthood qRT‐PCR: one‐day adults were placed on OP50 NGM FUdR plates at 20°C and 8 days later, 200 worms were harvested (Fig. 6d). RNA was isolated with Trizol (TRI REAGENT; Sigma, St. Louis, Missouri, United States), DNase‐treated, and cleaned over a column (RNA Clean & Concentrator^™^; ZYMO Research, Irvine, California, United States). First‐strand cDNA was synthesized in duplicate from each sample (Invitrogen, Carlsbad, California, United States, SuperScript III). SYBR green was used to perform qRT‐PCR (Applied Biosystems 7900, Thermo Fisher Scientific Inc., Waltham, Massachusetts, USA). For each primer set, a standard curve from genomic DNA accompanied the duplicate cDNA samples (Ewald *et al*., 2015). mRNA levels relative to N2 control were determined by normalizing to the number of worms and the geometric mean of three reference genes [*cdc‐42, pmp‐3,* and Y45F10D.4 (Hoogewijs *et al*., 2008)]. At least two biological replicates were examined for each sample. For statistical analysis, one‐sample *t*‐test, two‐tailed, hypothetical mean of 1 was used for comparison using Prism 4.0a software (GraphPad, La Jolla, California, United States).

### Fat quantification

Fixed Oil Red O (ORO) staining: Worms were synchronized by hypochlorite treatment and day 1 adult worms were fixed. ORO staining of fixed animals and quantification was performed.

Triglyceride quantification: Mixed‐stage worms were collected and prepared as described. Triglyceride levels were measured with the Triglyceride Colorimetric Assay Kit (#10010303; Cayman Chemical, Ann Arbor, MI, USA) and normalized to protein levels using BCA protein assay (Pierce Biotechnology, Thermo Fisher Scientific Inc., Waltham, Massachusetts, USA).

## Funding

This work was supported by grants from the Alzheimer's Association, National Institutes of Health (R21AG0339 and R01AG32042), and National Science Foundation (IOS08207) (CL) and a National Institutes of Health RCMI grant (G12‐RR03060‐25) to City College.

## Author contributions

CYE and CL designed the experiments, analyzed and interpreted the data. CYE, VM, and CL generated transgenic animals. CYE performed all experiments. CYE and CL wrote the manuscript in consultation with VM.

## Conflict of interest

The authors have no competing interests to declare.

## Supporting information


**Fig. S1** The APL‐1 effects on lifespan correlates with biomarkers of aging.

**Fig. S2** The effects on lifespan of APL‐1 overexpressing lines does not correlate with fat content.

**Fig. S3** Longevity of P*snb‐1*::APL‐1 animals depends on *hsf‐1, daf‐16,* but not on the unfolded protein response.

**Table S1** Hypodermal APL‐1 increases lifespan.

**Table S2** APL‐1 mediated lifespan extension requires DAF‐16 and DAF‐12.

**Table S3** APL‐1 longevity requires functional APL‐1 and is additive to reduced Insulin/IGF‐1 and reduced germstem cell number mediated longevity.

**Table S4** Hypodermal APL‐1 longevity by LIN‐14.
