# Supplementary figures and images for: Alzheimer‐related protein APL‐1 modulates lifespan through heterochronic gene regulation in Caenorhabditis elegans
Source: Aging Cell. 2016 Aug 24;15(6):1051–62. doi: 10.1111/acel.12509 (PMC5114704; doi:10.1111/acel.12509)

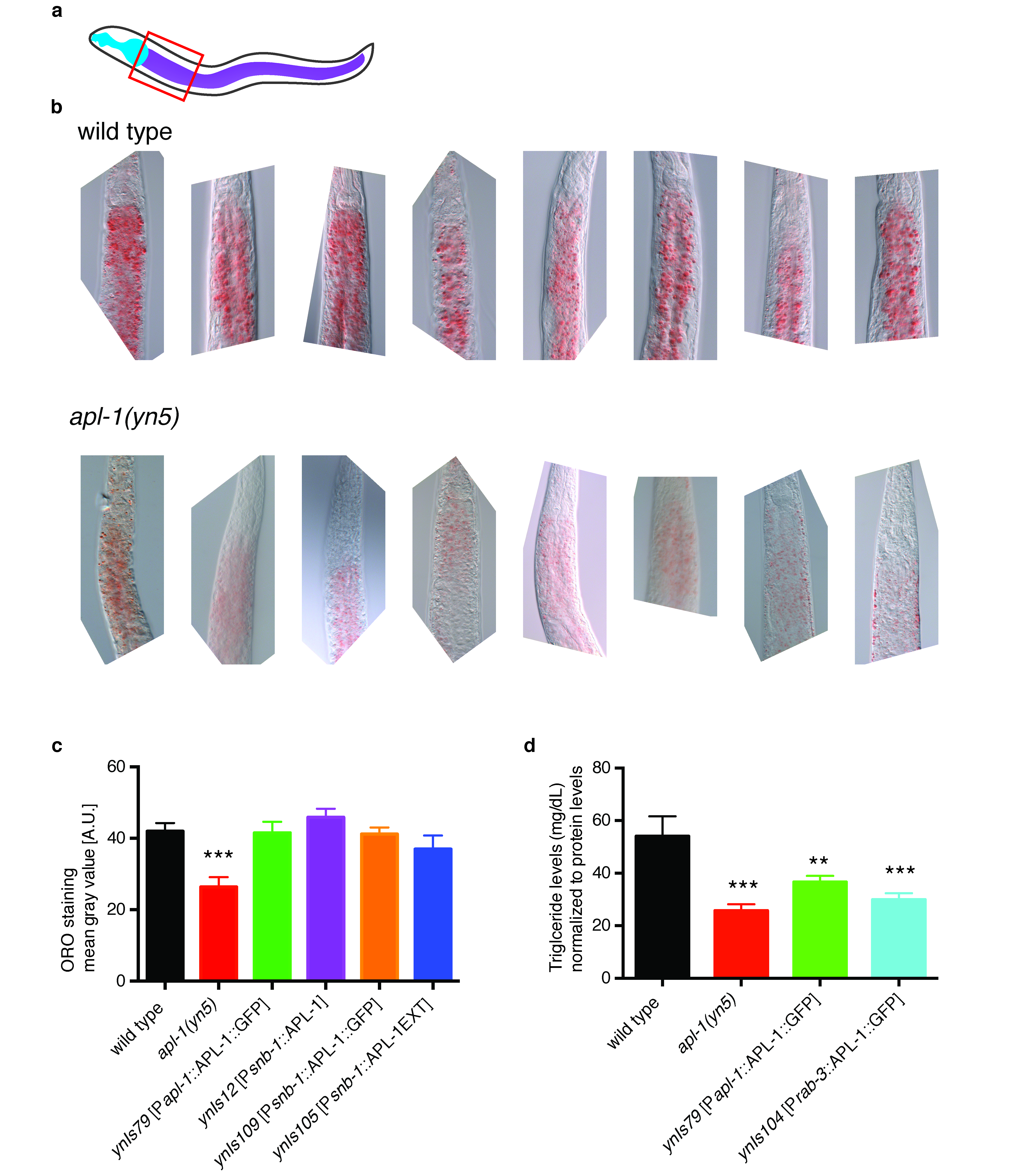

Supplement: Supplementary file 2 — Fig. S2 The effects on lifespan of APL‐1 overexpressing lines does not correlate with fat content. [file ACEL-15-1051-s002.tif]
