# Supplementary material for: Alzheimer‐related protein APL‐1 modulates lifespan through heterochronic gene regulation in Caenorhabditis elegans
Source: Aging Cell. 2016 Aug 24;15(6):1051–62. doi: 10.1111/acel.12509 (PMC5114704; doi:10.1111/acel.12509)
Supplement: Supplementary file 4 — Table S1 Hypodermal APL‐1 increases lifespan. [file ACEL-15-1051-s004.doc]

| **Table S1. Hypodermal APL-1 increases lifespan.** | | | | | |  |
| --- | --- | --- | --- | --- | --- | --- |
| Strain (Genotype) | Mean lifespan ± S.E.M.  [Days]* | 75^th^ percentile  [Days]** | N died from senescence/ Initial N*** | % Control(s) | P-Value against Control(s) | Fig |
| ***Heat-shock inducible APL-1 overexpression in all tissues (1 trial run in parallel)*** | | | | | |  |
| Maintained and assayed 15^o^C |  |  |  |  |  |  |
| wild type (N2) 15^o^C | 23.4 ± 0.3 | 25 | 77/90 |  |  | 1d |
| *ynIs14* [P*hsp-16.2*::APL-1] 15^o^C | 23.8 ± 0.3 | 25 | 90/104 | +2% | 0.1255 | 1d |
| Maintained at 15^o^C and upshifted to 25^o^C at the first day of adulthood | | | | | | |
| wild type (N2) 25^o^C | 12.5 ± 0.1 | 13 | 100/104 |  |  | 1c |
| *ynIs14* [P*hsp-16.2*::APL-1] 25^o^C | 16.2 ± 0.3 | 18 | 104/109 | +30% | <0.0001 | 1c |
| Maintained at 15^o^C and at the first day of adulthood one-time heat-shock 30^o^C for 3.5 hours, then assayed at 20^o^C **** | | | | | | |
| wild type (N2) 20^o^C single heat-shock | 21.9 ± 0.2 | 22 | 126/135 |  |  | 1a |
| *ynIs14* [P*hsp-16.2*::APL-1] 20^o^C single heat-shock | 23.1 ± 0.3 | 25 | 122/135 | +5% | <0.0001 | 1a |
| Maintained at 15^o^C and at the first day of adulthood repeated heat-shock each 30^o^C for 2 hours, assayed at 20^o^C | | | | | | |
| wild type (N2) 20^o^C repeated heat-shock | 22.1 ± 0.2 | 22 | 103/105 |  |  | 1b |
| *ynIs14* [P*hsp-16.2*::APL-1] 20^o^C repeated heat-shock | 24.8 ± 0.3 | 27 | 128/135 | +12% | <0.0001 | 1b |
| ***Neuronal and hypodermal APL-1 overexpression (1 trial run in parallel)*** | | | | | |  |
| wild type (N2) | 21.1 ± 0.3 | 23 | 91/100 |  |  | 1g |
| *ynEx214* {P*ceh-36*::APL-1::GFP} chemo. n. | 14.7 ± 0.3 | 16 | 72/84 | -30% | <0.0001 | 1g |
| *ynEx212* {P*mec-4*::APL-1::GFP} touch n. | 14.1 ± 0.3 | 16 | 67/80 | -33% | <0.0001 |  |
| *ynEx213* {P*mec-4*::APL-1::GFP} touch n. | 13.2 ± 0.3 | 14 | 51/65 | -37% | <0.0001 |  |
| *ynIs113* [P*mec-4*::APL-1::GFP] touch n. | 14.9 ± 0.3 | 16 | 83/94 | -29% | <0.0001 | 1h |
| *ynEx234* {P*col-10*::APL-1::GFP} hypodermis | 24.1 ± 0.5 | 28 | 52/66 | +14% | <0.0001 |  |
| ***Non-functional APL-1 overexpression driven by the* snb-1 *promoter (1 trial run in parallel)*** | | | | | | |
| non-transgenic siblings (from *ynEx237*) | 22.3 ± 0.5 | 25 | 79/105 |  |  | 2h |
| *ynEx236* {P*snb-1*::APL-1(E371K)::GFP} (line 1) | 22.9 ± 0.7 | 27 | 43/48 | +3% | 0.3849 | 2h |
| *ynEx237* {P*snb-1*::APL-1(E371K)::GFP} (line 2) | 20.9 ± 0.4 | 22 | 82/112 | -6% | 0.0202 | 2h |
| *ynEx238* {P*snb-1*::APL-1(E371K)::GFP} (line 3) | 22.0 ± 0.3 | 25 | 125/151 | -1% | 0.2021 | 2h |
| *ynIs109* [P*snb-1*::APL-1::GFP] | 27.1 ± 0.5 | 31 | 125/154 | +22% | <0.0001 | 2h |
| *ynEx214* {P*ceh-36*::APL-1::GFP} chemo. n. | 16.9 ± 0.2 | 19 | 92/121 | -24% | <0.0001 |  |
| *ynIs113* [P*mec-4*::APL-1::GFP] touch n. | 17.5 ± 0.2 | 19 | 106/128 | -22% | <0.0001 |  |
| ***Somatic gonad APL-1 overexpression driven by the* fln-1 *promoter (1 trial run in parallel)*** | | | | | | |
| non-transgenic siblings | 23.4 ± 0.4 | 27 | 106/125 |  |  | 3a |
| *ynEx242* {P*fln-1*::APL-1::GFP} (line 1) | 23.1 ± 0.3 | 25 | 118/135 | -1% | 0.1825 | 3a |
| *ynEx243* {P*fln-1*::APL-1::GFP} (line 2) | 22.9 ± 0.2 | 25 | 154/172 | -2% | 0.3678 | 3a |
| *ynEx244* {P*fln-1*::APL-1::GFP} (line 3) | 23.3 ± 0.2 | 25 | 150/172 | 0% | 0.5009 | 3a |
| ***Non-functional APL-1 overexpression driven by the* snb-1 *promoter and Somatic gonad APL-1 overexpression driven by the* fln-1 *promoter (1 trial run in parallel)*** | | | | | | |
| non-transgenic siblings (from *yn32* line 3) | 22.6 ± 0.4 | 25 | 66/94 |  |  |  |
| *ynEx236* {P*snb-1*::APL-1(E371K)::GFP} | 21.3 ± 0.4 | 23 | 77/108 | -6% | 0.0598 |  |
| *ynEx238* {P*snb-1*::APL-1(E371K)::GFP} | 22.7 ± 0.4 | 25 | 92/103 | 0% | 0.6428 |  |
| *ynEx242* {P*fln-1*::APL-1::GFP} | 23.2 ± 0.7 | 25 | 27/35 | +3% | 0.5118 |  |
| *ynEx243* {P*fln-1*::APL-1::GFP} | 22.7 ± 0.4 | 23 | 88/109 | 0% | 0.8730 |  |
| *ynEx244* {P*fln-1*::APL-1::GFP} | 24.1 ± 0.4 | 28 | 109/129 | +7% | 0.0328 |  |
| ***Hypodermal APL-1 overexpression driven by the* col-10 *promoter (1 trial run in parallel)*** | | | | | | |
| non-transgenic siblings | 23.1 ± 0.3 | 25 | 95/104 |  |  | 3b |
| *ynEx234* {P*col-10*::APL-1::GFP} | 27.0 ± 0.4 | 30 | 117/132 | +17% | <0.0001 | 3b |
| ***Hypodermal APL-1 overexpression driven by the* col-10 *promoter (1 trial run in parallel)*** | | | | | | |
| non-transgenic siblings | 21.9 ± 0.4 | 25 | 86/112 |  |  |  |
| *ynEx235* {P*col-10*::APL-1::GFP} | 24.3 ± 0.5 | 28 | 103/134 | +11% | <0.0001 |  |
| ***Hypodermal APL-1 overexpression driven by the* col-10 *promoter (1 trial run in parallel)*** | | | | | | |
| non-transgenic siblings | 22.6 ± 0.4 | 25 | 49/60 |  |  |  |
| *ynEx235* {P*col-10*::APL-1::GFP} | 25.4 ± 0.6 | 28 | 60/71 | +11% | <0.0001 |  |
| ***Hypodermal APL-1EXT overexpression driven by the* col-10 *promoter (1 trial run in parallel)*** | | | | | | |
| non-transgenic siblings | 21.5 ± 0.3 | 23 | 88/98 |  |  | 4e |
| *ynEx241* {P*col-10*::APL-1EXT} | 25.1 ± 0.4 | 28 | 96/108 | +17% | <0.0001 | 4e |
| ***Hypodermal APL-1EXT overexpression driven by the* col-10 *promoter (1 trial run in parallel)*** | | | | | | |
| wild type (N2) | 23.4 ± 0.2 | 23 | 71/83 |  |  |  |
| *ynEx241* {P*col-10*::APL-1EXT} | 26.1 ± 0.3 | 28 | 65/75 | +12% | <0.0001 |  |
| ***Hypodermal APL-1EXT overexpression driven by the* col-10 *promoter (1 trial run in parallel)*** | | | | | | |
| non-transgenic siblings (from *ynEx239*) | 23.5 ± 0.6 | 25 | 55/67 |  |  |  |
| *ynEx239* {P*col-10*::APL-1EXT} | 27.3 ± 0.5 | 30 | 80/90 | +16% | <0.0001 |  |
| *ynEx240* {P*col-10*::APL-1EXT} | 27.3 ± 0.5 | 30 | 65/73 | +16% | <0.0001 |  |
| *ynEx241* {P*col-10*::APL-1EXT} | 26.6 ± 0.5 | 28 | 50/59 | +13% | <0.0001 |  |

**Individual Adult Lifespans on NGM plates with FUDR.** All lifespan assays were performed on NGM plates containing 50 μg/ml FUDR. Unless otherwise indicated, lifespan assays were performed at 20^o^C. Individual lifespans are shown. *Is,* **[ ]** = integrated transgene; *Ex*, **{ }** = extrachromosomal transgene; **(N)** = number of animals; ***** Measured from L4 stage. To synchronize worm population, L4 animals were picked on NGM plates without FUDR and the next day adults were placed on NGM plates containing FUDR. ****** 75^th^ percentile is the age when a quarter of the population is still alive; ******* Total number of initial animals includes animals that died from senescence and censored animals that crawled off the plates, buried into the agar, bagged or exploded. n. = neuron; chemo. = chemosensory. P-values for lifespans were determined by Log-Rank test.
