# Supplementary material for: Alzheimer‐related protein APL‐1 modulates lifespan through heterochronic gene regulation in Caenorhabditis elegans
Source: Aging Cell. 2016 Aug 24;15(6):1051–62. doi: 10.1111/acel.12509 (PMC5114704; doi:10.1111/acel.12509)
Supplement: Supplementary file 5 — Table S2 APL‐1 mediated lifespan extension requires DAF‐16 and DAF‐12. [file ACEL-15-1051-s005.doc]

| **Table S2. APL-1 mediated lifespan extension requires DAF-16 and DAF-12.** | | | | | |  |
| --- | --- | --- | --- | --- | --- | --- |
| Strain (Genotype) | Mean lifespan ± S.E.M.  [Days]* | 75^th^ percentile  [Days]** | N died from senescence/ Initial N***, (T) | % Control(s) | P-Value against Control(s) | Fig |
| ***Controls*** | | | | | |  |
| wild type (N2) ^†^ | 14.2 ± 0.1 | 16 | 1275/2537(31) |  |  | 1e, 1f |
| *lon-2(e678) apl-1(yn10)/*  *dpy-8(e130)* | 14.5 ± 0.3 | 17 | 67/107 (1) | +2%^†^ | 0.0729^†^ |  |
| *ynIs107* [*Papl-1*::APL-1(E371K/D342C/S362C)::GFP] | 14.3 ± 0.3 | 16 | 105/203 (2) | +1%^†^ | 0.4766^†^ |  |
| *ynIs100* [*Papl-1*::APL-1(E371K)::GFP] 0.5x^§^ | 14.5 ± 0.3 | 15 | 100/153 (1) | +2%^†^ | 0.2481 ^†^ |  |
| *ynIs106* [*Papl-1*::APL-1EXT(E371K)] | 14.1 ± 0.2 | 15 | 115/144 (1) | -1%^†^ | 0.2481 ^†^ |  |
| *vsIs13* [*lin-15*(+)] | 14.2± 0.2 | 15 | 232/345 (3) | 0%^†^ | 0.2477^†^ |  |
| ***Endogenous Overexpression of full-length APL-1*** | | | | | |  |
| *ynIs86* [P*apl-1*::APL-1] 125x^§^ | 11.9 ± 0.2 | 13 | 151/240 (3) | -16%^†^ | <0.0001^†^ |  |
| *ynIs79* [P*apl-1*::APL-1::GFP] 182x^§^ | 11.7 ± 0.3 | 14 | 145/203 (3) | -18%^†^ | <0.0001^†^ | 1f |
| *apl-1(yn10)* {P*apl-1*::APL-1} | 11.9 ± 0.3 | 14 | 31/54 (1) | -16%^†^ | <0.0001^†^ |  |
| {P*apl-1*::APL-1} | 13.4 ± 0.7 | 13 | 17/50 (1) | -6%^†^ | 0.2225^†^ |  |
| ***Endogenous Overexpression of the Extracellular Domain of APL-1 (APL-1EXT)*** | | | | | |  |
| *apl-1(yn5)* 16x^§^ | 12.5 ± 0.1 | 14 | 341/563 (6) | -12%^†^ | <0.0001^†^ |  |
| *ynIs71* [P*apl-1*::APL-1EXT] | 11.7 ± 0.3 | 14 | 139/254 (3) | -18%^†^ | <0.0001^†^ |  |
| ***Pan-neural expression of APL-1 or GFP driven by* rab-3 *promoter*** | | | | | |  |
| *ynEx93* {P*rab-3*::APL-1::GFP } | 12.9 ± 0.5 | 14 | 29/119 (1) | -9%^†^ | 0.0352^†^ |  |
| *ynIs104* [P*rab-3*::APL-1::GFP] 8x^§^ | 10.9 ± 0.2 | 11 | 142/312 (3) | -23%^†^ | <0.0001^†^ | 1e |
| *ynIs91* [P*rab-3*::APL-1::GFP] | 12.1 ± 0.2 | 14 | 202/276 (3) | -15%^†^ | <0.0001^†^ |  |
| *ynIs91* [P*rab-3*::APL-1::GFP]; *vsIs13* [*lin-15*(+)] | 12.2 ± 0.3 | 14 | 71/152 (1) | -14%^†^ | <0.0001^†^ |  |
| *jsIs682* [P*rab-3*::GFP::RAB-3] | 13.3 ± 0.5 | 15 | 78/111 (1) | -6%^†^ | 0.18575^†^ |  |
| ***Overexpression of APL-1 driven by* snb-1 *promoter*** | | | | | |  |
| *ynIs12* [P*snb-1*::APL-1] 71x^§^ | 17.6 ± 0.2 | 20 | 309/746 (8) | +24%^†^ | <0.0001^†^ |  |
| *ynIs13* [P*snb-1*::APL-1] 17x^§^ | 17.2 ± 0.2 | 20 | 257/529 (5) | +21%^†^ | <0.0001^†^ |  |
| *ynIs12* [P*snb-1:*:APL-1]*; ynIs86* [P*apl-1*::APL-1] 205x^§^ | 17.7 ± 0.3 | 20 | 124/296 (3) | +25%^†^ | <0.0001^†^ |  |
| ***Overexpression of the Extracellular Domain of APL-1 driven by* snb-1 *promoter*** | | | | | |  |
| *ynEx65* {P*snb-1*::APL-1EXT} | 16.0 ± 0.3 | 17 | 59/120 (1) | +13%^†^ | <0.0001^†^ |  |
| *ynIs105* [P*snb-1*::APL-1EXT] | 17.7 ± 0.3 | 21 | 138/319 (3) | +25%^†^ | <0.0001^†^ |  |
| ***Overexpression of APL-1 in a* daf-16(mu38) *null background*** | | | | | |  |
| *daf-16(mu86)* | 13.1 ± 0.1 | 14 | 264/408 (5) | -8%^†^ | <0.0001^†^ |  |
| *daf-16(mu86); ynIs86* [P*apl-1*::APL-1] | 10.9 ± 0.2 | 12 | 91/120 (1) | -17%^◊^; -23%^†^ | <0.0001^◊^ |  |
| *daf-16(mu86); ynIs79* [P*apl-1*::APL-1::GFP] | 12.2 ± 0.3 | 15 | 66/103 (1) | -7%^◊^; -14%^†^ | 0.0217^◊^ |  |
| *daf-16(mu86); apl-1(yn5)* | 10.9 ± 0.3 | 13 | 86/186 (2) | -17%^◊^; -23%^†^ | <0.0001^◊^ |  |
| *daf-16(mu86); apl-1(yn10)* {P*apl-1*::APL-1} | 11.4 ± 0.3 | 13 | 96/122 (1) | -12%^◊^; -20%^†^; -4%^0^ | <0.0001^◊^  0.3398^0^ |  |
| *daf-16(mu86); ynIs104* [P*rab-3*::APL-1::GFP] | 9.5 ± 0.3 | 10 | 80/125 (2) | -27%^◊^; -33%^†^ | <0.0001^◊^ |  |
| *daf-16(mu86); ynIs12* [P*snb-1*::APL-1] | 13.2 ± 0.2 | 15 | 222/319 (3) | -1%^◊^; -7%^†^ | 0.9848^◊^ |  |
| *daf-16(mu86); ynIs13* [P*snb-1*::APL-1] | 13.1 ± 0.2 | 16 | 190/265 (3) | 0%^◊^; -8%^†^ | 0.9852^◊^ |  |
| *daf-16(mu86); ynIs105* [P*snb-1*::APL-1EXT] | 13.2 ± 0.2 | 15 | 109/191 (3) | -1%^◊^; -7%^†^ | 0.8374^◊^ |  |
| ***Overexpression of APL-1 in a* daf-2(e1370) *reduction-of-function background*** | | | | | |  |
| *daf-2(e1370)* | 37.8 ± 0.6 | 44 | 224/327 (3) | +166%^†^ | <0.0001^†^ |  |
| *daf-2(e1370) {*P*apl-1*::APL-1} | 38.3 ± 1.3 | 46 | 70/111 (1) | +1%^; +169%^†^ | 0.1038^ |  |
| *daf-2(e1370); ynIs79* [P*apl-1*::APL-1::GFP] | 35.7 ± 1.0 | 44 | 85/124 (1) | -6%^; +151%^†^ | 0.0801^ |  |
| *daf-2(e1370); apl-1(yn10)* {P*apl-1*::APL-1} | 24.4 ± 0.6 | 31 | 148/253 (2) | -35%^; +72%^†^ | <0.0001^ |  |
| *daf-2(e1370); apl-1(yn5)* | 35.8 ± 1.8 | 45 | 30/61 (1) | -6%^; +151%^†^ | 0.2482^ |  |
| *daf-2(e1370); ynIs12* [P*snb-1*::APL-1] | 43.0 ± 1.1 | 50 | 82/101 (1) | +14%^; +203%^†^ | <0.0001^ |  |
| *daf-2(e1370); ynIs104* [P*rab-3*::APL-1::GFP] | 26.2 ± 1.0 | 31 | 97/176 (1) | -31%^; +85%^†^ | <0.0001^ |  |
| ***Overexpression of APL-1 and DAF-16*** | | | | | |  |
| *zIs356* [DAF-16::GFP] | 17.9 ± 0.2 | 19 | 173/242 (2) | +26%^†^ | <0.0001^†^ |  |
| *zIs356*; *ynIs79* [P*apl-1*::APL-1::GFP] | 17.8 ± 0.5 | 24 | 156/204 (2) | -1%^#^; +25%^†^ | 0.7151^#^ |  |
| *zIs356*; *apl-1(yn5)* | 13.8 ± 0.2 | 16 | 157/288 (2) | -23%^#^; -3%^†^ | <0.0001^#^; 0.6867^†^ |  |
| *zIs356*; *ynIs104* [P*rab-3*::APL-1::GFP] | 15.8 ± 0.2 | 18 | 106/153 (1) | -12%^#^; +11%^†^ | <0.0001^#^ |  |
| *zIs356*; *ynIs12* [P*snb-1*::APL-1] | 20.2 ± 0.3 | 21 | 155/376 (2) | +13%^#^; +42%^†^ | <0.0001^#^ |  |
| ***Overexpression of APL-1 in a* daf-12(m20) *mutant background*** | | | | | |  |
| *daf-12(m20)* | 11.6 ± 0.2 | 14 | 298/487 (4) | -18%^†^ | <0.0001^†^ |  |
| *daf-12(m20)*; *ynIs12* [P*snb-1*::APL-1] | 11.3 ± 0.2 | 14 | 241/402 (3) | -3%@; -20%^†^ | 0.5240@;<0.0001^†^ |  |
| *daf-12(m20)*; *ynIs105* [P*snb-1*::APL-1EXT] | 10.8 ± 0.3 | 11 | 60/102 (1) | -7%@; -24%^†^ | 0.0565@;<0.0001^†^ |  |

**Cumulative Adult Lifespan on MYOB plates without FUDR.** All lifespan assays were performed on MYOB plates without FUDR. Unless otherwise indicated, lifespan assays were performed at 20^o^C. All lifespans are shown in cumulative form, since individual trials do not differ significantly from cumulative and since experimental animals were always assayed with their proper control and wild-type animals. *Is,* **[ ]** = integrated transgene; *Ex*, **{ }** = extrachromosomal transgene; **(N)** = number of animals observed; **(T)** = number of independent trials the experiment was performed; ***** Measured from L4 stage. ****** 75^th^ percentile is the age when a quarter of the population is still alive; ******* Total number of initial animals includes animals that died from senescence and censored animals that crawled off the plates, buried into the agar, bagged or exploded; **†** = wild type (N2); **0** = *apl-1(yn10)* {P*apl-1*::APL-1}; **◊** = *daf-16(mu86)*, all APL-1 overexpression animals in a *daf-16(mu86)* null background are significantly different than wild type by P<0.0001; **^** = *daf-2(e1370)*, all APL-1 overexpression animals in a *daf-2(e1370)* background are significantly different than wild type by P<0.0001; @ = *daf-12(m20)*. *P*-values for lifespans were determined by Log-Rank test. *lon-2(e678)* and *dpy-8(e130)* are flanking genetic markers of *apl-1*. ^§^ = fold overexpression of APL-1 as determined by western blot analysis. For *ynIs12* and *ynIs13* P*snb-1*::APL-1 was co-injected *lin-15*(+) into *lin-15*(-) animals. When transgenes were integrated the *lin-15*(-) mutation was outcrossed.
