# Supplementary material for: Alzheimer‐related protein APL‐1 modulates lifespan through heterochronic gene regulation in Caenorhabditis elegans
Source: Aging Cell. 2016 Aug 24;15(6):1051–62. doi: 10.1111/acel.12509 (PMC5114704; doi:10.1111/acel.12509)
Supplement: Supplementary file 6 — Table S3 APL‐1 longevity requires functional APL‐1 and is additive to reduced Insulin/IGF‐1 and reduced germstem cell number mediated longevity. [file ACEL-15-1051-s006.doc]

| **Table S3. APL-1 longevity requires functional APL-1 and is additive to reduced Insulin/IGF-1 and reduced germstem cell number mediated longevity.** | | | | | |  |
| --- | --- | --- | --- | --- | --- | --- |
| Strain (Genotype) | Mean lifespan ± S.E.M.  [Days]* | 75^th^ percentile  [Days]** | N died from senescence/ Initial N***, (T) | % Control(s) | P-Value against Control(s) | Fig |
| ***Overexpression of APL-1, human APP, or SNB-1 driven by* snb-1 *promoter*** | | | | | |  |
| *jsIs1* [P*snb-1*::SNB-1::GFP] used as control | 13.9 ± 0.3 | 17 | 98/114 (1) |  |  | 2c |
| *ynIs109* [P*snb-1*::APL-1::GFP] | 17.8 ± 0.3 | 18 | 72/108 (1) | +28% | <0.0001 | 2c |
| *dvEx371* [P*snb-1*::humanAPP_751_] #1 | 13.7 ± 0.3 | 15 | 80/102 (1) | -1% | 0.5376 | 2c |
| *dvEx372* [P*snb-1*::humanAPP_751_] #2 | 13.5 ± 0.2 | 15 | 85/106 (1) | -3% | 0.0741 | 2c |
| *dvIs62* [P*snb-1*::humanTDP-43] | 5.3 ± 0.2 | 5 | 101/103 (1) | -62% | <0.0001 | 2c |
| *P*-value and % mean lifespan change are relative to *jsIs1* [P*snb-1*::SNB-1::GFP] control | | | | | | |
| ***RNAi knockdown of* apl-1 *or* daf-12** | | | | | |  |
| RNAi of L4440 on wild type (N2) | 16.9 ± 0.6 | 19 | 39/54 (1) |  |  | 2d |
| RNAi of *apl-1* on wild type (N2) | 15.1 ± 0.4 | 16 | 49/73 (1) | -11% | 0.0040 | 2d |
| RNAi of *daf-12* on wild type (N2) | 11.8 ± 0.5 | 12 | 44/77 (1) | -30% | <0.0001 |  |
| RNAi of L4440 on *ynIs13* [P*snb-1*::APL-1]* | 19.3 ± 0.6 | 22 | 39/74 (1) | +14% | 0.0479 | 2d |
| RNAi of *apl-1* on *ynIs13* [P*snb-1*::APL-1] | 14.4 ± 0.4 | 16 | 40/72 (1) | -15% | 0.0162 *<0.0001 | 2d |
| RNAi of *daf-12* on *ynIs13* [P*snb-1*::APL-1] | 11.3 ± 0.3 | 12 | 52/91 (1) | -33% | <0.0001 *<0.0001 |  |
| *P*-value and % mean lifespan change are relative to RNAi of L4440 on wild type (N2) and to * RNAi of L4440 on *ynIs13* [P*snb-1*::APL-1] | | | | | | |
| ***Lifespan of double transgenic APL-1 overexpression driven by* apl-1 *and/or* snb-1 *promoter*** | | | | | |  |
| wild type (N2) | 14.7 ± 0.4 | 16 | 78/101 |  |  | 2i |
| *ynIs86* [P*apl-1*::APL-1] | 11.8 ± 0.2 | 13 | 71/102 | -19% | <0.0001 | 2i |
| *ynIs12* [P*snb-1*::APL-1] | 18.2 ± 0.5 | 21 | 60/79 | +24% | <0.0001 | 2i |
| *ynIs86;ynIs12* | 17.8 ± 0.4 | 21 | 77/98 | +21% | <0.0001 | 2i |
| *P*-value and % mean lifespan change are relative to wild type (N2) | | | | | | |
| **daf-16 *is* *required for P*snb-1*::APL-1EXT* *induced longevity*** | | | | | |  |
| wild type (N2) | 13.5 ± 0.6 | 16 | 54/72 (1) |  |  | 5a |
| *ynIs105* [P*snb-1*::APL-1EXT] | 17.8 ± 0.4 | 21 | 80/90 (1) | +32% | <0.0001 | 5a |
| *daf-16(mu86)** | 12.6 ± 0.3 | 14 | 43/61 (1) | -7% | 0.2252 | 5a |
| *daf-16(mu86); ynIs105* [P*snb-1*::APL-1EXT] | 12.3 ± 0.3 | 14 | 52/65 (1) | -9% | 0.2053  *0.8576 | 5a |
| *P*-value and % mean lifespan change are relative to wild type (N2) and * to *daf-16(mu86)* | | | | | | |
| **daf-12 *is* *required for P*snb-1*::APL-1EXT* *induced longevity*** | | | | | |  |
| wild type (N2) | 15.0 ± 0.4 | 17 | 62/77 (1) |  |  | 5b |
| *ynIs105* [P*snb-1*::APL-1EXT] | 18.4 ± 0.4 | 21 | 55/71 (1) | +23% | <0.0001 | 5b |
| *daf-12(m20)** | 11.5 ± 0.2 | 14 | 80/96 (1) | -23% | <0.0001 | 5b |
| *daf-12(m20); ynIs105* [P*snb-1*::APL-1EXT] | 10.6 ± 0.3 | 11 | 63/76 (1) | -29% | <0.0001  *0.225 | 5b |
| *P*-value and % mean lifespan change are relative to wild type (N2) and * to *daf-12(m20)* | | | | | | |
| ***Lifespan at 25^o^C starting as L1 for APL-1 overexpression in a* glp-1(e2141ts) *mutant background*** | | | | | | |
| wild type (N2) | 11.7 ± 0.2 | 13 | 68/87 (1) |  |  | 5c |
| *ynIs71* [P*apl-1*::APL-1EXT] | 10.8 ± 0.3 | 12 | 58/76 (1) | -8% | 0.0037 |  |
| *ynIs105* [P*snb-1*::APL-1EXT] | 11.5 ± 0.3 | 12 | 35/49 (1) | -2% | 0.2213 |  |
| *glp-1(e2141)** | 15.0 ± 0.4 | 16 | 69/69 (1) | +28% | <0.0001 | 5c |
| *glp-1(e2141); ynIs71* [P*apl-1*::APL-1EXT] | 14.6 ± 0.4 | 19 | 76/76 (1) | +25% | *0.4962 |  |
| *glp-1(e2141); ynIs105* [P*snb-1*::APL-1EXT] | 20.3 ± 0.4 | 23 | 116/127 (1) | +74% | <0.0001  *<0.0001 | 5c |
| *P*-value and % mean lifespan change are relative to wild type (N2) and * to *glp-1(e2141)* | | | | | | |
| ***APL-1 overexpression in a* daf-2(e1370) *mutant background*** | | | | | | |
| wild type (N2) | 13.3 ± 0.5 | 15 | 50/69 (1) |  |  | 5d |
| *daf-2(e1370)** | 34.1 ± 1.0 | 41 | 84/106 (1) | +157% | <0.0001 | 5d |
| *daf-2(e1370); ynIs12* [P*snb-1*::APL-1] | 43.0 ± 1.1 | 50 | 82/101 (1) | +223% | <0.0001  *<0.0001 | 5d |
| *P*-value and % mean lifespan change are relative to wild type (N2) and relative to * *daf-2(e1370)* | | | | | | |
| ***Lifespan at 25^o^C starting as L1 for APL-1 overexpression in a* tcer-1(tm1452ts) *mutant background*** | | | | | |  |
| wild type (N2) | 12.3 ± 0.7 | 13 | 77/87 (1) |  |  |  |
| *tcer-1(tm1452)* * | 14.0 ± 0.5 | 15 | 53/102 (1) | +14% | 0.0448 |  |
| *ynIs109* [P*snb-1*::APL-1::GFP] | 16.1 ± 0.5 | 19 | 61/63 (1) | +31% | <0.0001  *<0.0001 |  |
| *tcer-1(tm1452); ynIs109* [P*snb-1*::APL-1::GFP] | 8.1 ± 0.2 | 8 | 87/90 (1) | -34% | <0.0001  *<0.0001 |  |
| *P*-value and % mean lifespan change are relative to wild type (N2) and * to *tcer-1(tm1452)* | | | | | | |
| ***RNAi knockdown of candidate genes required for P*snb-1*::APL-1EXT* *induced longevity*** | | | | | |  |
| RNAi of L4440 on wild type (N2) | 14.2 ± 0.3 | 16 | 117/229 (2) |  |  | S3g |
| RNAi of *hsf-1* on wild type (N2) | 9.5 ± 0.2 | 10 | 89/147 (1) | -33% | <0.0001 | S3g |
| RNAi of *skn-1* on wild type (N2) | 12.6 ± 0.5 | 15 | 66/129 (1) | -11% | 0.0247 | S3g |
| RNAi of *daf-16* on wild type (N2) | 11.4 ± 0.4 | 14 | 60/115 (1) | -20% | <0.0001 | S3g |
| RNAi of *kri-1* on wild type (N2) | 10.5 ± 0.3 | 11 | 66/126 (1) | -26% | <0.0001 | S3g |
| RNAi of *tcer-1* on wild type (N2) | 13.3 ± 0.4 | 15 | 67/125 (1) | -6% | 0.0796 | S3g |
| RNAi of L4440 on *ynIs105* [P*snb-1*::APL-1EXT] | 17.0 ± 0.4 | 21 | 79/130 (1) | +20% | <0.0001 | S3g |
| RNAi of *hsf-1* on *ynIs105* [P*snb-1*::APL-1EXT] | 9.9 ± 0.1 | 11 | 88/121 (1) | -30% | <0.0001, *0.0874 | S3g |
| RNAi of *skn-1* on *ynIs105* [P*snb-1*::APL-1EXT] | 14.1 ± 0.2 | 16 | 98/120 (1) | -1% | 0.3616, *0.1473 | S3g |
| RNAi of *daf-16* on *ynIs105* [P*snb-1*::APL-1EXT] | 11.7 ± 0.2 | 14 | 97/118 (1) | -18% | <0.0001, *0.7527 | S3g |
| RNAi of *kri-1* on *ynIs105* [P*snb-1*::APL-1EXT] | 11.5 ± 0.2 | 14 | 72/119 (1) | -19% | <0.0001, *0.0301 | S3g |
| RNAi of *tcer-1* on *ynIs105* [P*snb-1*::APL-1EXT] | 15.2 ± 0.3 | 16 | 84/129 (1) | +7% | 0.1075, *0.0024 | S3g |
| *P*-value and % mean lifespan change are relative to RNAi of L4440 on wild type (N2) and * RNAi of corresponding gene on wild type (N2) | | | | | | |
| ***Overexpression of APL-1EXT lacking heparin binding domain*** | | | | | |  |
| wild type (N2) | 16.6 ± 0.4 | 18 | 63/74 (1) |  |  |  |
| *ynIs108* [P*apl-1*::APL-1(ΔH, ΔE2)::GFP] | 15.8 ± 0.4 | 18 | 55/62 (1) | -5% | 0.1908 |  |
| *P*-value and % mean lifespan change are relative to wild type (N2) | | | | | | |

**Individual Adult Lifespans on MYOB plates without FUDR.** All lifespan assays were performed on MYOB plates without FUDR. Unless otherwise indicated, lifespan assays were performed at 20^o^C. Individual lifespans are shown *Is,* **[ ]** = integrated transgene; *Ex*, **{ }** = extrachromosomal transgene; **(N)** = number of animals observed; **(T)** = number of independent trials the experiment was performed; ***** Measured from L4 stage. ****** 75^th^ percentile is the age when a quarter of the population is still alive; ******* Total number of initial animals includes animals that died from senescence and censored animals that crawled off the plates, buried into the agar, bagged or exploded; P-values for lifespans were determined by Log-Rank test.. L4440 is empty vector control for RNAi.
