# Supplementary material for: Alzheimer‐related protein APL‐1 modulates lifespan through heterochronic gene regulation in Caenorhabditis elegans
Source: Aging Cell. 2016 Aug 24;15(6):1051–62. doi: 10.1111/acel.12509 (PMC5114704; doi:10.1111/acel.12509)
Supplement: Supplementary file 7 — Table S4 Hypodermal APL‐1 longevity by LIN‐14. [file ACEL-15-1051-s007.doc]

| **Table S4. Hypodermal APL-1 longevity by LIN-14** | | | | | |  |
| --- | --- | --- | --- | --- | --- | --- |
| Strain (Genotype) | Mean lifespan ± S.E.M.  [Days]* | 75^th^ percentile  [Days]** | N died from senescence/ Initial N***, (T) | % Control(s) | P-Value against Control(s) | Fig |
| ***Longevity from adulthood-specific* lin-14(RNAi) *and hypodermal APL-1 are not additive*** | | | | | |  |
| wild type (N2) EV | 23.9 ± 0.2 | 25 | 65/70 (1) |  |  | 6a |
| wild type (N2) *lin-14(RNAi)* | 27.3 ± 0.3 | 29 | 71/79 (1) | +14% | <0.0001 | 6a |
| *ynEx234* {P*col-10*::APL-1::GFP} EV * | 27.7 ± 0.3 | 29 | 84/89 (1) | +16% | <0.0001 | 6a |
| *ynEx234* {P*col-10*::APL-1::GFP} *lin-14(RNAi)* | 28.4 ± 0.4 | 29 | 76/80 (1) | +19% | <0.0001  *0.0937 | 6a |
| *ynIs109* [P*snb-1*::APL-1::GFP] EV ^$^ | 28.1 ± 0.3 | 29 | 121/129 (1) | +18% | <0.0001 | 6a |
| *ynIs109* [P*snb-1*::APL-1::GFP] *lin-14(RNAi)* | 28.4 ± 0.3 | 32 | 127/131 (1) | +19% | <0.0001  ^$^0.5140 | 6a |
| *P*-value and % mean lifespan change are relative to wild type (N2) EV,* to [P*col-10*::APL-1::GFP] line 1 EV, and ^$^ to *ynIs109* [P*snb-1*::APL-1::GFP]EV | | | | | | |
| ***Longevity of* lin-14 *mutants and hypodermal APL-1 are not additive***  *(all strains were raised at 15^o^C and shifted to 20^o^C for lifespan assay)* | | | | | |  |
| wild type (N2) | 22.2 ± 0.3 | 24 | 64/71 (1) |  |  | 6b |
| *ynIs109* [P*snb-1*::APL-1::GFP] | 26.2 ± 0.3 | 29 | 103/111 (1) | +18% | <0.0001 | 6b |
| *lin-14(n179)** | 25.5 ± 0.4 | 27 | 73/80 (1) | +15% | <0.0001 | 6b |
| *ynIs109* [P*snb-1*::APL-1::GFP]; *lin-14(n179)* | 25.5 ± 0.3 | 27 | 109/117 (1) | +15% | <0.0001  *0.8673 | 6b |
| *zaIs2* [P*lin-14*::LIN-14::GFP] ^$^ | 21.1 ± 0.4 | 25 | 67/76 (1) | -5% | 0.2013 | 6b |
| *ynIs109* [P*snb-1*::APL-1::GFP]; *zaIs2* [P*lin-14*::LIN-14::GFP] | 17.9 ± 0.3 | 20 | 85/95 (1) | -19% | <0.0001  ^$^<0.0001 | 6b |
| *P*-value and % mean lifespan change are relative to wild type (N2) EV, * to *lin-14(n179)*, and ^$^ to *zaIs2* [P*lin-14*::LIN-14::GFP] | | | | | | |
| ***Longevity of* lin-14 mutants *and hypodermal APL-1 are not additive***  *(all strains were raised at 15^o^C and shifted to 20^o^C for lifespan assay)* | | | | | |  |
| wild type (N2) | 23.9 ± 0.2 | 25 | 92/97 (1) |  |  |  |
| *ynIs109* [P*snb-1*::APL-1::GFP] | 27.8 ± 0.2 | 29 | 107/113 (1) | +16% | <0.0001 |  |
| *lin-14(n179)** | 26.8 ± 0.3 | 29 | 106/117 (1) | +12% | <0.0001 |  |
| *ynIs109* [P*snb-1*::APL-1::GFP]; *lin-14(n179)* | 26.6 ± 0.2 | 29 | 109/118 (1) | +11% | <0.0001  *0.5373 |  |
| *zaIs2* [P*lin-14*::LIN-14::GFP] ^$^ | 23.4 ± 0.3 | 25 | 117/128 (1) | -2% | 0.4661 |  |
| *ynIs109* [P*snb-1*::APL-1::GFP]; *zaIs2* [P*lin-14*::LIN-14::GFP] | 20.6 ± 0.2 | 22 | 119/128 (1) | -14% | <0.0001  ^$^<0.0001 |  |
| *P*-value and % mean lifespan change are relative to wild type (N2) EV, * to *lin-14(n179)*, and ^$^ to *zaIs2* [P*lin-14*::LIN-14::GFP] | | | | | | |
| ***Longevity of hypodermal APL-1 is suppressed by higher LIN-14 protein levels*** | | | | | |  |
| non-transgenic siblings (from *ynEx234*) | 21.5 ± 0.2 | 22 | 76/85 (1) |  |  | 6c |
| *ynEx234* {P*col-10*::APL-1::GFP} | 26.2 ± 0.6 | 28 | 60/66 (1) | +22% | <0.0001 | 6c |
| *zaIs2* [P*lin-14*::LIN-14::GFP]*** | 19.6 ± 0.2 | 20 | 60/65 (1) | -9% | <0.0001 | 6c |
| *zaIs2* [P*lin-14*::LIN-14::GFP] *ynEx234* {P*col-10*::APL-1::GFP} | 19.2 ± 0.3 | 20 | 58/66 (1) | -11% | <0.0001  *0.4221 | 6c |
| *P*-value and % mean lifespan change are relative to non-transgenic siblings (from *ynEx234*) and * to *zaIs2* [P*lin-14*::LIN-14::GFP] | | | | | | |

**Individual Adult Lifespans on NGM plates with FUDR.** All lifespan assays were performed on NGM plates containing 50 μg/ml FUDR. Unless otherwise indicated, lifespan assays were performed at 20^o^C. Individual lifespans are shown. *Is,* **[ ]** = integrated transgene; *Ex*, **{ }** = extrachromosomal transgene; **(N)** = number of animals; **(T)** = number of independent trials the experiment was performed; ***** Measured from L4 stage. To synchronize worm population, L4 animals were picked on NGM plates without FUDR and the next day adults were placed on NGM plates containing FUDR and if indicated RNAi bacteria (i.e. adulthood specific knockdown for all RNAi lifespans excluding developmental effects). ****** 75^th^ percentile is the age when a quarter of the population is still alive; ******* Total number of initial animals includes animals that died from senescence and censored animals that crawled off the plates, buried into the agar, bagged or exploded. *P*-values for lifespans were determined by Log-Rank test. EV stands for empty vector plasmid L4440 and is used as a control for RNAi.
